# Real-time mapping of gapless 24-hour surface PM_10_ in China

**DOI:** 10.1093/nsr/nwae446

**Published:** 2024-12-09

**Authors:** Xutao Zhang, Ke Gui, Hengheng Zhao, Nanxuan Shang, Zhaoliang Zeng, Wenrui Yao, Lei Li, Yu Zheng, Hujia Zhao, Yurun Liu, Yucong Miao, Yue Peng, Ye Fei, Fugang Li, Baoxin Li, Hong Wang, Zhili Wang, Yaqiang Wang, Huizheng Che, Xiaoye Zhang

**Affiliations:** State Key Laboratory of Severe Weather and Key Laboratory of Atmospheric Chemistry of China Meteorological Administration, Chinese Academy of Meteorological Sciences, Beijing 100081, China; State Key Laboratory of Severe Weather and Key Laboratory of Atmospheric Chemistry of China Meteorological Administration, Chinese Academy of Meteorological Sciences, Beijing 100081, China; State Key Laboratory of Severe Weather and Key Laboratory of Atmospheric Chemistry of China Meteorological Administration, Chinese Academy of Meteorological Sciences, Beijing 100081, China; State Key Laboratory of Severe Weather and Key Laboratory of Atmospheric Chemistry of China Meteorological Administration, Chinese Academy of Meteorological Sciences, Beijing 100081, China; Institute of Artificial Intelligence for Meteorology, Chinese Academy of Meteorological Sciences, Beijing 100081, China; State Key Laboratory of Severe Weather and Key Laboratory of Atmospheric Chemistry of China Meteorological Administration, Chinese Academy of Meteorological Sciences, Beijing 100081, China; State Key Laboratory of Severe Weather and Key Laboratory of Atmospheric Chemistry of China Meteorological Administration, Chinese Academy of Meteorological Sciences, Beijing 100081, China; State Key Laboratory of Severe Weather and Key Laboratory of Atmospheric Chemistry of China Meteorological Administration, Chinese Academy of Meteorological Sciences, Beijing 100081, China; Institute of Atmospheric Environment, China Meteorological Administration, Shenyang 110166, China; State Key Laboratory of Severe Weather and Key Laboratory of Atmospheric Chemistry of China Meteorological Administration, Chinese Academy of Meteorological Sciences, Beijing 100081, China; Plateau Atmospheric and Environment Key Laboratory of Sichuan Province, College of Atmosphere Sciences, Chengdu University of Information Technology, Chengdu 610225, China; State Key Laboratory of Severe Weather and Key Laboratory of Atmospheric Chemistry of China Meteorological Administration, Chinese Academy of Meteorological Sciences, Beijing 100081, China; State Key Laboratory of Severe Weather and Key Laboratory of Atmospheric Chemistry of China Meteorological Administration, Chinese Academy of Meteorological Sciences, Beijing 100081, China; National Meteorological Information Center, Beijing 100081, China; China Global Atmosphere Watch Baseline Observatory, Xining 810001, China; Greenhouse Gas and Carbon Neutral Key Laboratory of Qinghai Province, Xining 810001, China; China Global Atmosphere Watch Baseline Observatory, Xining 810001, China; Greenhouse Gas and Carbon Neutral Key Laboratory of Qinghai Province, Xining 810001, China; State Key Laboratory of Severe Weather and Key Laboratory of Atmospheric Chemistry of China Meteorological Administration, Chinese Academy of Meteorological Sciences, Beijing 100081, China; State Key Laboratory of Severe Weather and Key Laboratory of Atmospheric Chemistry of China Meteorological Administration, Chinese Academy of Meteorological Sciences, Beijing 100081, China; Institute of Artificial Intelligence for Meteorology, Chinese Academy of Meteorological Sciences, Beijing 100081, China; State Key Laboratory of Severe Weather and Key Laboratory of Atmospheric Chemistry of China Meteorological Administration, Chinese Academy of Meteorological Sciences, Beijing 100081, China; State Key Laboratory of Severe Weather and Key Laboratory of Atmospheric Chemistry of China Meteorological Administration, Chinese Academy of Meteorological Sciences, Beijing 100081, China

**Keywords:** PM_10_, real time, seamless retrieval, interpretable machine learning, dust storm

## Abstract

Large-scale mapping of surface coarse particulate matter (PM_10_) concentration remains a key focus for air quality monitoring. Satellite aerosol optical depth (AOD)-based data fusion approaches decouple the non-linear AOD–PM_10_ relationship, enabling high-resolution PM_10_ data acquisition, but are limited by spatial incompleteness and the absence of nighttime data. Here, a gridded visibility-based real-time surface PM_10_ retrieval (RT-SPMR) framework for China is introduced, addressing the gap in seamless hourly PM_10_ data within the 24-hour cycle. This framework utilizes multisource data inputs and dynamically updated machine-learning models to produce 6.25-km gridded 24-hour PM_10_ data. Cross-validation showed that the RT-SPMR model's daily retrieval accuracy surpassed prior studies. Additionally, through rolling iterative validation experiments, the model exhibited strong generalization capability and stability, demonstrating its suitability for operational deployment. Taking a record-breaking dust storm as an example, the model proved effective in tracking the fine-scale evolution of the dust intrusion process, especially in under-observed areas. Consequently, the operational RT-SPMR framework provides comprehensive real-time capability for monitoring PM_10_ pollution in China, and has the potential to improve the accuracy of dust storm forecasting models by enhancing the PM_10_ initial field.

## Teaser

This paper introduces a real-time surface PM_10_ retrieval framework for China, enabling gapless 24-hour PM_10_ monitoring. It has the potential for improving the accuracy of dust storm forecasting models by enhancing the PM_10_ initial field.

## INTRODUCTION

After almost a decade of absence, multiple extreme dust storm events reemerged in northern China between 2021 and 2023, presenting considerable environmental challenges [1,2]. The increasing frequency of these events underscores the urgent need to enhance dust monitoring methodologies. Surface coarse particulate matter (PM_10_) concentration is a principal pollutant in dust storms and a crucial indicator for evaluating air quality and environmental impacts [3,4]. Accurate monitoring of PM_10_ concentrations is vital, as it provides essential data on the severity and expanse of dust storms, as well as their potential hazards to the environment and human health. Real-time and precise PM_10_ data are fundamental for enhancing early warning systems, devising effective mitigation strategies and mitigating the adverse effects of dust storms, thereby improving the overall efficiency of public health and environmental protection measures.

Despite the pivotal role of ground-based monitoring stations in PM_10_ data acquisition, their sparse distribution and the limitations of localized measurements lead to considerable observational gaps, particularly during expansive dust storm events [1,5,6]. To address these gaps, external data sources have been integrated, with advanced algorithms being used to combine satellite spectral products (e.g. aerosol optical depth (AOD)) with a variety of ancillary data [7–12]. However, AOD-based PM_10_ retrievals, irrespective of whether derived using polar-orbiting or geostationary satellites, are often limited by spatial incompleteness caused by cloud interference and the absence of nighttime data attributable to the unavailability of nighttime AOD products [13–15]. Moreover, these methods rely on reanalysis meteorological data inputs (e.g. the fifth generation European Centre for Medium-Range Weather Forecasts atmospheric reanalysis of the global climate), which have temporal acquisition delays that limit the real-time data acquisition capabilities of the resulting PM_10_ retrieval products. Therefore, current research is focused primarily on constructing historical data sets rather than on obtaining real-time data retrievals. A review of existing research revealed the current lack of a real-time PM_10_ retrieval strategy capable of providing all-weather, 24-hour, spatially continuous coverage.

In contrast, surface visibility (SV), a crucial measure of atmospheric transparency, is generally less constrained by these factors and has demonstrated potential for estimating surface fine PM (PM_2.5_) concentrations [13,16]. However, existing studies predominantly focus on using SV to estimate site-scale PM_2.5_ concentrations, with limited exploration of its applicability for PM_10_. Given that PM_10_ has a weaker effect on light scattering compared to PM_2.5_ for the same mass [17], estimating PM_10_ concentrations from SV presents greater challenges. Therefore, addressing the non-linear relationship between SV and PM_10_ concentration is essential for the effective retrieval of spatiotemporal gapless PM_10_ concentrations from SV data.

**Figure 1.**
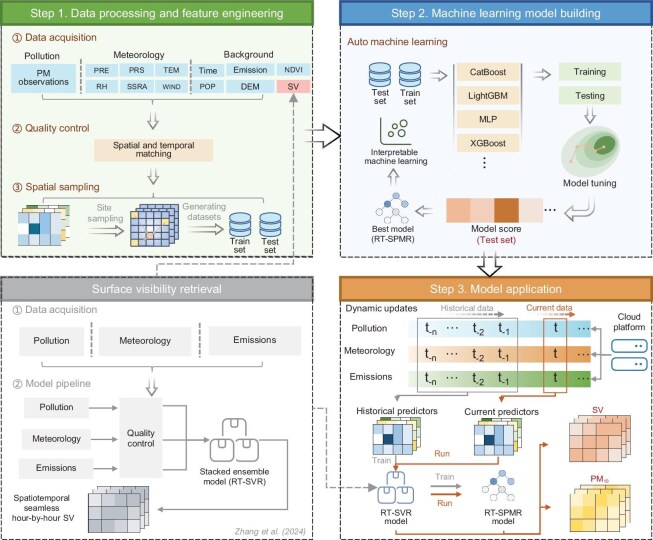
Construction and operational workflow of the RT-SPMR framework. This workflow involves three main steps: Step 1: data integration, which processes previously developed SV data [18] and other multisource data. Step 2: model building, which employs automated ML methods to develop an optimal, self-iterating model. Step 3: model deployment, which fine-tunes the model using dynamically updated data from the past two years and retrieves hourly PM_10_ concentrations for the most recent day.

In this study, we introduce the first real-time surface PM_10_ retrieval framework (RT-SPMR) for China (Fig. 1), which employs a newly developed gridded SV data set as a fundamental parameter [18]. The RT-SPMR framework, based on interpretable automated machine learning (ML) with dynamic updates, utilizes multisource data fusion to achieve real-time retrieval of gridded PM_10_ data in China with high spatial/temporal resolution (6.25 km/1 h). The real-time availability of spatially gapless and continuous 24-hour PM_10_ data markedly enhances the capability to track PM_10_ pollution events, such as dust storms, and has substantial potential for improving the initial fields of dust forecasting models used in China.

## RESULTS

### Performance of the RT-SPMR model

Considering the requirements for computational efficiency and retrieval accuracy in real-time operational processes, this study employed an automated ML (AutoML) technique (see Materials and Methods) to identify the optimal RT-SPMR model from six candidate ML models (Fig. 1). The AutoML framework enables multi-model and multi-parameter tuning, allowing us to select a model that optimally balances both accuracy and runtime efficiency, while avoiding the subjective biases of arbitrary model selection. We conducted iterative training and tuning of each candidate model using a training set from 2020 to 2021, followed by hindcast validation (HV) using an independent validation set for 2022, representative of a real-world application scenario. Model performance was assessed using three metrics: the coefficient of determination (*R*^2^), root mean square error (RMSE) and computational time cost.

Table S1 lists the prediction performance of the various ML models used for the HV experiments following parameter tuning. The results indicate that the deep learning model (i.e. Multi-Layer Perceptron (MLP)) is less effective and more time consuming for PM_10_ retrieval. In contrast, tree-based models demonstrate notable advantages when handling tabular data [19]. Specifically, the PM_10_ values estimated using the CatBoost, LightGBM and ExtraTrees models exhibit higher accuracy for independent data sets, with *R*^2^ (RMSE) values of 0.69 (35.1 μg·m^−3^), 0.68 (35.7 μg·m^−3^) and 0.67 (36.5 μg·m^−3^), respectively. Although the PM_10_ retrieval accuracy among the three gradient-boosting tree-based models is comparable, the performance of CatBoost is better owing to its superior Graphic Processing Unit (GPU) acceleration capabilities that make it fastest in real-time operation. Consequently, CatBoost was selected as the RT-SPMR model used in this study.

**Figure 2.**
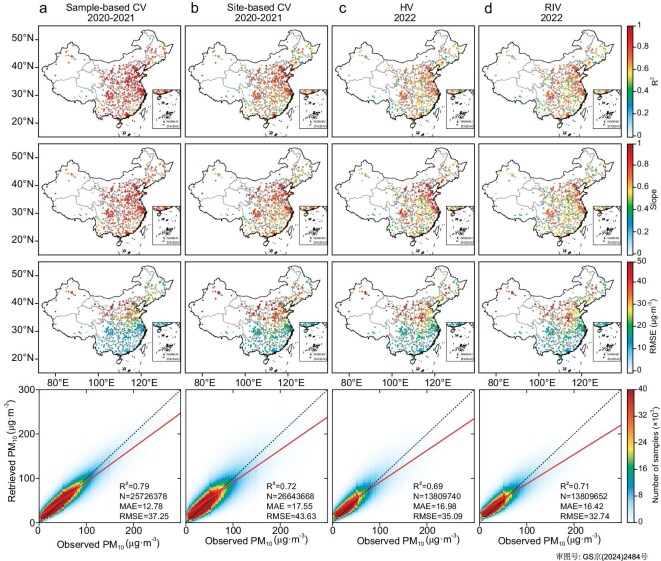
Performance of the RT-SPMR model. The first three rows show the spatial distribution of statistical results between observed PM_10_ and (a) sample-based 5-fold CV, (b) site-based 5-fold CV, (c) HV and (d) RIV-estimated PM_10_ at each site. The final row presents density scatterplots between the observed PM_10_ and the estimated PM_10_ from the aforementioned validation methods. Note that all statistics here are generated with hourly temporal resolution. Note that data for Taiwan are not available in this study.

In addition to the HV (using the 2022 validation set), we conducted an integrated evaluation of the RT-SPMR performance based on the training set from 2020–2021 using two widely adopted validation approaches: sample-based 5-fold cross-validation (CV) and site-based 5-fold CV [20]. The PM_10_ estimates obtained from the sample-based CV, tested on ∼25.7 × 10^6^ data pairs, exhibit reasonable agreement with the observations, i.e. *R*^2^ = 0.79, RMSE = 37.25 μg·m^−3^ (Fig. 2a). The results for site-based CV (*R*^2 ^= 0.72, RMSE = 43.63 μg·m^−3^) are slightly worse than those for sample-based CV; however, >60% of sites have an *R*^2^ value of >0.7 (Fig. 2b). For the HV experiment using one-year continuous PM_10_ observations (∼13.8 × 10^6^ samples; Fig. 2c), the RT-SPMR model still exhibits strong generalization capabilities, albeit with slight degradation in performance (*R*^2^ = 0.69, RMSE = 35.1 μg·m^−3^; also see Table S1). Spatially, the three statistical indicators (*R*^2^, slope and RMSE) obtained from the three different validation methods (sample-based CV, site-based CV, and HV) show similar distribution patterns (Fig. 2a–c) but with slight differences in magnitude. Overall, the model performs well in regions such as the North China Plain (NCP), East China and the Sichuan Basin, where dense networks of PM_10_ observation sites are available for model training. In contrast, the model performs poorly on the Tibetan Plateau, in northwestern China and in northeastern China, which are regions with sparsely distributed observation sites. Additionally, in these regions, the bias in the SV data also contributes to bias in PM_10_ retrievals [18,21,22]. Notably, compared with the southern region, the model has larger RMSE in the northern region, primarily owing to the impact of irregularly occurring dust storm events. The sharp fluctuations in PM_10_ concentrations during dust storms might increase the model bias for the overall PM_10_ retrieval sample. In the southern region, where it is less likely that dust aerosols from the desert will be transported, the moderate RMSE is mainly attributed to the effect of more regular anthropogenic PM_10_ pollution.

Figures S1–S3 show the performance of the RT-SPMR model on the hourly scale based on the sample-based CV, site-based CV, and HV, respectively. All three evaluation results indicate that the RT-SPMR model demonstrates strong robustness at each hour, despite some hourly dependent differences in performance. Overall, the model performs slightly better during the daytime (08:00–17:00, China Standard Time, CST) than at night (18:00–07:00 CST). For instance, for HV, the model has an average R^2^ of 0.71 (0.69) and an RMSE of 33.44 μg·m^−3^ (36.26 μg·m^−3^) during the daytime (nighttime). This could be attributable to the more complex relationships between PM_10_, SV and ambient humidity during nighttime. During daytime, the warming effect caused by solar radiation might enable the model to achieve better fitting capabilities under low humidity conditions.

### Comparison with prior studies

We conducted a comprehensive survey of previous studies of national-scale quantitative PM_10_ retrievals in China. Table S2 summarizes the comparison between our study and prior studies in terms of including the dominant predictors, spatiotemporal resolution, accuracy and updating capability. We found that previous studies almost exclusively relied on satellite signals (e.g. top of atmosphere) or AOD products as primary input predictors. The spatial resolution of these products was generally 4–10 km, dictated primarily by the spatial resolution of the satellite product. In terms of temporal resolution, past PM_10_ retrievals were typically available only on the daily scale owing to the unavailability of satellite-based AOD products at night. The only exception involved the use of geostationary satellite products (e.g. Himawari-8), which can acquire hourly products during specific daytime hours. Real-time, gapless PM_10_ data availability is crucial for timely tracking of PM_10_ pollution events. Unfortunately, no studies to date have provided real-time updates, mainly owing to delays in accessing reanalysis meteorological fields and satellite products.

In contrast, the RT-SPMR model achieves real-time, high-spatial-resolution, 24-hour gapless PM_10_ mapping using real-time accessible hourly SV instead of the widely used AOD as the key predictor, by integrating real-time meteorological data from the China Meteorological Administration Land Data Assimilation System (CLDAS). Beyond the advantages in terms of spatiotemporal resolution and real-time updating, our PM_10_ product is comparable with, or even better than, previous PM_10_ products in terms of evaluation accuracy. For example, on the daily scale, our product has higher *R²* and lower RMSE values for both the sample-based and the site-based CV methods. These results underscore the advantages of the gridded SV-based retrieval strategy in filling the gaps in hourly PM_10_ data from satellite remote sensing over a 24-hour cycle and ensuring real-time accessibility. More detailed spatial comparisons across different temporal scales, using the ChinaHighAirPollutants (CHAP) data set [7] as reference, are discussed in the following sections.

### Stability testing of the dynamically updated RT-SPMR model

During the operational runs, we eschewed the use of a static, one-time trained RT-SPMR model in favor of a real-time iterative updating strategy to enhance the accuracy of real-time PM_10_ retrievals. To evaluate the stability of this dynamically updated RT-SPMR model, we conducted an additional rolling iterative validation (RIV). In the RIV, we updated the RT-SPMR model in real-time using sliding windows of one and two years, utilizing the data within each window for model training and employing data from the subsequent 24 hours of the first day after the window for validation.

**Figure 3.**
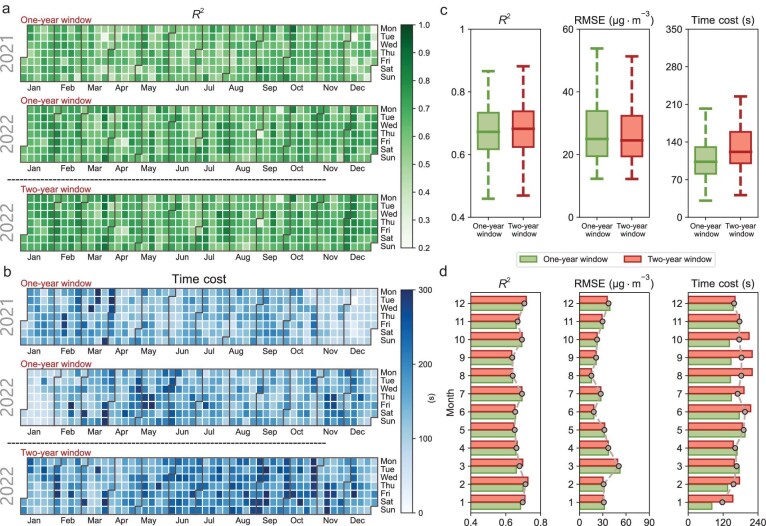
Performance of the RT-SPMR model with rolling updates in operational settings. (a) Daily performance (expressed as *R*^2^) of the RT-SPMR model when trained using a one-year rolling window and a two-year rolling window. Note that the daily *R*^2^ is calculated between the PM_10_ retrievals and the observations for the day following a sliding window of one or two years. (b) Same as (a) but for the time cost associated with model training for both the one-year and the two-year rolling windows. (c) Boxplot of *R*^2^, RMSE and time cost for the two rolling training methods. To ensure consistency in sample size, only the results from 2022 are compared. (d) Monthly performance of the RT-SPMR model, including *R*^2^, RMSE and time cost, when trained using a one-year rolling window and a two-year rolling window.

We computed daily statistics for the 24-hourly sample sizes and obtained the day-by-day dynamics of the statistical indicators (Fig. 3). Our findings indicate that the RT-SPMR model, iteratively updated with a one-year (two-year) sliding window, demonstrated stable performance on daily and monthly time scales for both 2021 and 2022 (2022). As illustrated in Fig. 3c, using the unified 2022 validation set, the RT-SPMR model with a two-year sliding window outperformed its one-year counterpart, achieving daily averaged *R*^2^ and RMSE values of 0.68 and 29.13 μg·m^−3^, respectively, compared with averaged values of *R*^2 ^= 0.67 and RMSE = 30.03 μg·m^−3^ for the one-year window. Even when considering hourly data, the model trained with a two-year sliding window (*R*^2^ = 0.71, RMSE = 32.74 μg·m^−3^, see Fig. 2d) outperformed the model trained with a one-year sliding window (*R*^2^ = 0.68, RMSE = 35.64 μg·m^−3^, not shown here). Furthermore, the two-year sliding window showed superior performance relative to the HV test (Fig. 2c) in the RIV results (see Fig. 2d). These results suggest that the iteratively updated RT-SPMR model effectively captures updates from multiple data sources in real time, enhancing the generalization capability of the model and mitigating overfitting.

In terms of computational efficiency, the daily average computation time for the two-year window was marginally higher than that for the one-year window by ∼30 s (Fig. 3c and d). This minor difference in time cost is negligible when considering the overall lag time of the input data. Consequently, considering the higher accuracy and computational efficiency provided by the two-year window, we opted for a two-year sliding window for iterative updates of the RT-SPMR model during the final operational run. Testing on workstations equipped with GeForce RTX 3090 GPU devices revealed that the total time required for the entire RT-SPMR framework—from data processing to model updating and performing the one hourly PM_10_ retrieval task—was ∼45–50 min. This timeframe includes the waiting period for CLDAS and the retrieval of SV data. Such a comprehensive operationalization framework ensures the real-time accessibility of hourly PM_10_ data.

To further confirm the advantage of the dynamically updated RT-SPMR model over the static model (i.e. without dynamic updating), we analyzed the performance of both during a typical dust event from 20 April to 22 April 2022. Figure S4 presents the time series of observed PM_10_ concentrations, as well as HV and RIV (i.e. the model for two-year window) estimates for four representative sites (Tongliao, Hohhot, Tianjin and Qinhuangdao) during this event. The results demonstrate that the RIV model consistently outperforms the HV model at all four sites, highlighting the benefits of iterative updating in enhancing model accuracy. In addition to the individual case analysis, the hourly time series of observed and RIV-estimated PM_10_ concentrations at several representative sites in Baotou, Tianjin, Chongqing and Guangzhou for the entire year of 2022 demonstrate the robustness of the dynamically updated RT-SPMR model. As shown in Fig. S5, the model accurately reproduces both low and high values, as well as trends in areas with varying levels of pollution.

### Diurnal cycle of gapless PM_10_ in China

**Figure 4.**
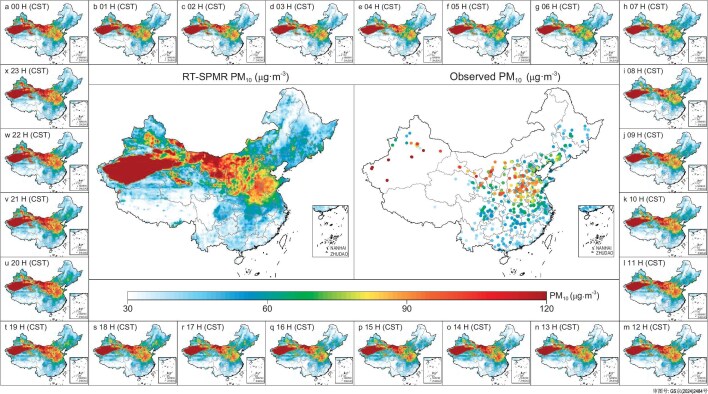
Spatial distributions of retrieval PM_10_. (a–x) Multiyear (2020–2022) averaged hourly PM_10_ maps. Central panels show the multiyear annual average PM_10_ from the RT-SPMR model (shading) and observations (points). Note that data for Taiwan are not available in this study.

Figure 4 illustrates the distribution of the annual mean gapless PM_10_ products retrieved by the RT-SPMR model, trained on data from 2020 to 2022, together with the corresponding annual mean PM_10_ observations. The results demonstrate a high degree of consistency between the spatial distributions of the RT-SPMR-derived annual mean PM_10_ and the ground-based PM_10_ observations. On the national scale, the gapless PM_10_ product effectively captures the dynamic distribution of PM_10_ in regions lacking observational data. Spatially, the annual average PM_10_ hotspots in China are located predominantly in dust source areas, including the Taklamakan Desert, Qaidam Basin and Gobi Desert (GD). Notably, extensive regions of elevated PM_10_ concentrations are observed downstream of the GD, extending from northwestern Inner Mongolia to the Shandong Peninsula. These areas with high levels of PM_10_ concentration are influenced by both the springtime dust transport belt from the GD and the wintertime anthropogenic emissions in northern China (Fig. S6). Additionally, we compared the annual and seasonal PM_10_ data retrieved by the RT-SPMR model with the CHAP data set for each year from 2020 to 2022. The results indicate strong correspondence between the two data sets across different years and seasons, in terms of both the spatial distribution of PM_10_ and the concentration level (Fig. S7).

Figures 4 and S8 present the 24-h-cycle maps of gapless PM_10_ retrievals and PM_10_ observations, averaged over the period 2020–2022. The results indicate that the RT-SPMR model accurately captures the diurnal variation characteristics of PM_10_ in China, which are characterized primarily by higher nighttime concentrations compared with daytime levels. We conducted spatiotemporal sampling of the gridded PM_10_ data set based on the geographic locations of the PM_10_ observation sites, and then performed nationwide site averaging of the sampled data with observed data to analyze the PM_10_ diurnal variations across different years (Fig. S9). Overall, the diurnal variation patterns of PM_10_ extracted by the RT-SPMR model are highly consistent with the observations, despite bias toward hourly dependence. The multiyear averaged data show that PM_10_ concentrations peaked in the morning between 09:00 and 12:00 CST, while a trough occurred between 14:00 and 18:00 CST. The morning peak was associated mainly with increased anthropogenic activities and poor conditions for vertical atmospheric dispersion, whereas the afternoon trough was linked with increased solar radiation and improved vertical dispersion conditions [23,24]. It is noteworthy that on an annual average scale, our model tends to overestimate the observed values by ∼2–3 μg·m^−3^ during 12:00–20:00 CST. In contrast, it tends to underestimate the observed values by 2–3 μg·m^−3^ during 21:00–11:00 CST. This over- or underestimation, which is particularly pronounced around noon, coincides with periods of rapid atmospheric changes driven by increased solar radiation. Seasonally, while the diurnal bias pattern remains consistent, the magnitude of these biases varies (Fig. S10). For instance, the model exhibits a more pronounced negative bias relative to observations before noon in spring, while the positive bias after noon is more evident in autumn and winter. These results suggest that the RT-SPMR model still faces challenges in accurately capturing the complex coupling between anthropogenic emissions and meteorological conditions, especially during periods of rapidly shifting atmospheric dynamics.

### Drivers of hourly and intra-annual variations in regional PM_10_

To better understand the model's decision-making process and the role of each predictor in the model output, we first performed a process-scale attribution analysis using the SHapley Additive exPlanations (SHAP) approach. Figure S4 shows the hourly SHAP values for each predictor at four representative sites along the transport path during a typical dust event from 20 April to 22 April 2022. The site-scale SHAP analysis reveals the dominant contribution of SV-driven PM_10_ changes during the extreme PM_10_ event. The increase in positive SHAP values for SV coincides with the emergence of PM_10_ peaks (accompanied by an increase in positive SHAP values for wind), while the shift to negative SHAP values drives the decline in PM_10_ concentrations later in the event. This further underscores the importance of SV's contribution to the model and its critical role in improving modeling performance.

**Figure 5.**
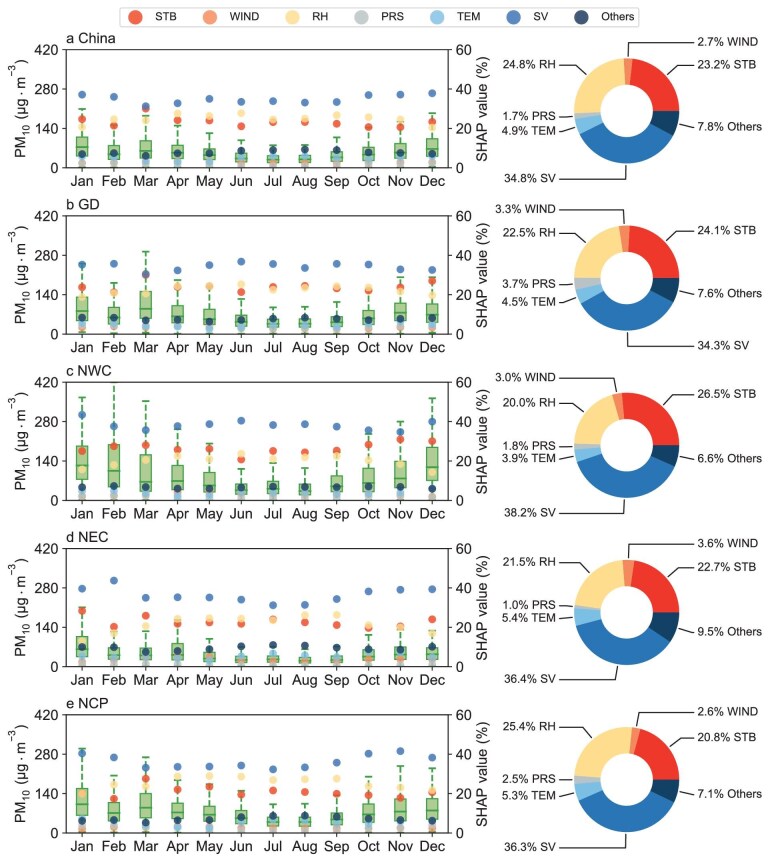
Decomposition of drivers in PM_10_ retrieval in China and the typical dust source areas. Monthly average PM_10_ values (boxplot), SHAP values (scatterplot) for each feature in the model, and their relative importance (pie chart) in (a) China, (b) GD, (c) NWC, (d) NEC and (e) NCP. See Fig. S11 for the geographic location of each subregion. Here, we grouped all the features into seven categories: WIND (U- and V-wind components), RH (relative humidity), PRS (surface pressure), TEM (temperature), SV (surface visibility), STB (longitude, latitude, elevation and temporal features) and other (NDVI, population, sand fraction and PM_10_ emission inventory).

Figure 5 illustrates the intra-annual contributions of seven predictors, as categorized in this study, to the SHAP values of PM_10_ outputs in the RT-SPMR model across China and in four representative regions. These regions include two major dust source areas, i.e. Northwest China (NWC) and the GD, and the downstream regions affected most by dust aerosols, i.e. the NCP and Northeast China (NEC). The geographical locations of these regions are shown in Fig. S11.

Overall, across China, SV emerges as the primary driver, accounting for 34.8% of the total contributions to all PM_10_ retrieval samples during the study period, followed by relative humidity (RH; 24.8%) and spatiotemporal background (STB; 23.2%). On a monthly scale, SV's contribution is dominant throughout the year, followed by RH and STB, whose contributions are relatively close. This indicates that SV plays a pivotal role in overall PM_10_ modeling, yet this process is also susceptible to the synergistic influence of RH and STB. Notably, the magnitude of SV's contribution increases during months with elevated PM_10_ levels (October to February), which correspond to periods of high anthropogenic emissions, suggesting that SV typically plays a key role in retrieving high anthropogenic PM_10_. Regionally, while the top three dominant factors remained consistent with the national level, differences were observed in the monthly variation of their relative contributions. In GD, SV's monthly contributions generally exhibit an inverse relationship to PM_10_ levels, with slight enhancements in summer. In NWC, although SV remains the dominant driver, STB's influence surpasses that of RH. In NEC and NCP, driven by anthropogenic coarse particulate emissions, SV dominates in autumn and winter, but its dominance diminishes in spring and summer as RH strengthens (with an increase in RH's contribution). In summary, SV, RH and STB are identified as the three most critical predictors influencing PM_10_ retrieval in the RT-SPMR model. However, their intra-annual importance varies, primarily because of the combined effects of anthropogenic activities, dust storm events and meteorological conditions.

### Fine-scale tracking of dust storm processes

Dust storms, as a major source of coarse particles in the atmosphere, have considerable impact on surface PM_10_ concentrations, and pose a serious threat both to the environment and to public health. Fine-scale monitoring plays a crucial role in capturing the details and dynamics of dust storms, but it faces challenges when implemented over wide geographical areas. While satellite observations offer a broad perspective for dust storm monitoring, interference from clouds makes it challenging to achieve continuous, hourly and full-coverage monitoring of related quantitative parameters, e.g. AOD and dust optical depth, during both daytime and nighttime.

**Figure 6.**
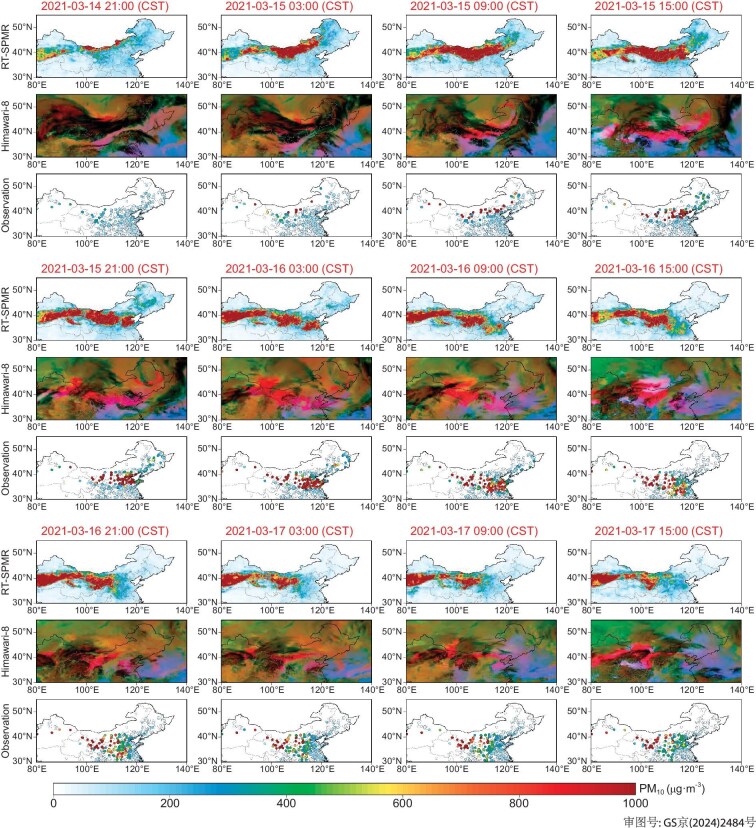
Tracking a large-scale dust storm incursion process using gapless PM_10_ from the RT-SPMR model. The 6-hourly evolution of dust plumes as revealed by gapless PM_10_ retrievals, Himawari-8 dust RGB composite images and PM_10_ observations during a dust storm event that occurred from 21:00 (CST) on 14 March to 15:00 (CST) on 17 March 2021. Note that data for Taiwan are not available in this study.

To evaluate the applicability of the RT-SPMR model in real-time dust storm monitoring, we selected a severe dust storm event that started on 14 March 2021 in northern China, which was the most intense dust event in that region in nearly two decades [1]. We compared the 6-hourly PM_10_ retrievals with synchronous red, green and blue (RGB) composite dust imagery from the Himawari-8 satellite and ground-based observational data (Fig. 6). The results indicate that the RT-SPMR model successfully reproduces the entire invasion process of this dust storm. It originated from southern Mongolia, crossed the border between Inner Mongolia and northwestern China, and gradually moved eastward under the influence of a large-scale Mongolian cyclone, impacting regions such as the NCP, NEC and Shandong Peninsula, leading to a notable surge in surface PM_10_ concentrations. The spatiotemporal dynamics of the PM_10_ concentrations retrieved by the RT-SPMR model show high consistency with the RGB dust imagery in terms of affected areas, and the PM_10_ levels also closely match ground-based observations. On the daily scale, our PM_10_ retrievals exhibit strong spatial correspondence with those based on satellite AOD (Fig. S12). These findings demonstrate that the RT-SPMR model can effectively capture the real-time dynamic changes of PM_10_ in the blind spots of geostationary satellite images and ground-based observation gaps, thereby robustly supporting fine-scale monitoring of dust storm processes.

## CONCLUSION AND DISCUSSION

This study proposed a novel RT-SPMR framework based on high-precision SV data that we had developed previously. Utilizing multisource data inputs and automatic ML models, this framework enables real-time, gapless, 24-hour surface PM_10_ mapping with high spatial/temporal resolution (6.25 km/1 h) across China. The CV results demonstrate that the RT-SPMR model outperforms existing satellite-based PM_10_ retrieval methods on the daily scale. More importantly, this approach addresses the considerable gap in hourly PM_10_ data within the 24-h cycle in current satellite-based retrieval strategies. Dynamic iterative updates of the RT-SPMR model using a defined two-year rolling window revealed that it meets the requirements for computational efficiency and accuracy stability in operational use. Application of the RT-SPMR model to an extreme dust storm event showcased its ability to accurately reproduce hourly PM_10_ dynamics in regions not covered by either geostationary satellite-derived dust imagery or surface observations. Considering the real-time retrieval capability of the RT-SPMR model, the resulting real-time PM_10_ outputs contribute to improving the fine-grained monitoring of PM_10_ pollution in China, particularly for dust storms, and provide crucial data for developing accurate pollution control policies and early warning systems. This, in turn, will support more effective environmental management and public health decision-making. Furthermore, integrating the real-time PM_10_ outputs into the initial fields of dust models is expected to further improve the accuracy of future dust storm forecasts in China [25].

Overall, the RT-SPMR framework proposed in this study successfully overcomes the limitations of current satellite-based PM_10_ retrieval strategies, marking a substantial advancement in gapless PM_10_ monitoring. Nevertheless, the RT-SPMR model still exhibits certain biases that future research could address through the following improvements. (i) Improving the accuracy of SV products to better represent the SV–PM_10_ response relationships within the model. For example, during SV retrieval [18], the accuracy of the PM background field in western China can be enhanced by applying deep learning and modeling data to reduce SV uncertainty. (ii) Incorporating more PM_10_ observations and additional predictors, such as geostationary satellite signals, to enhance the generalization capability of the model. (iii) Developing advanced retrieval models that account for temporal cyclic mechanisms and spatial effects, such as incorporating information from surrounding grids, to more accurately capture rapid spatiotemporal variations in PM_10_ concentrations. These improvements are anticipated to further refine the accuracy of PM_10_ retrievals, thereby providing more reliable and detailed data support for atmospheric environmental monitoring.

## MATERIALS AND METHODS

### Multisource data

This study used multisource data sets to generate target values (PM_10_ observations) and a matrix of predictors, including gridded SV data, meteorological fields, anthropogenic emission inventories and other ancillary data, spanning 2020–2022. Table S3 and Text S1 provide an overview of all multisource input data sets used in this study and the data processing procedures, including quality control and spatiotemporal matching. We eventually split the complete data set into two parts: data from 2020 to 2021 are used for model training (referred to as the training set), while data from 2022 are used for HV to assess the predictive capability of the model (referred to as the validation set).

### RT-SPMR framework with AutoML

The RT-SPMR framework utilizes multisource data inputs and dynamically updated interpretable AutoML models for the retrieval module, enabling output of gridded PM_10_ data with high spatial/temporal resolution (6.25 km/1 h). Overall, the process of construction of the RT-SPMR framework, illustrated in Fig. 1, comprises three modules: (i) multisource data processing and feature engineering, (ii) automated ML model building and tuning, and (iii) model deployment and operational workflow. The detailed processes involved in each module are summarized in Text S2.

### Explainable ML

Explaining the influence of each feature on model outputs can enhance our understanding of the model mechanisms, especially in terms of elucidating the decisions made by the model for PM_10_ retrieval at different time points. In this study, we employed the SHAP method to conduct an interpretable analysis of the decision-making process of the ML model. The SHAP method represents a pioneering approach in the field of ML interpretability, utilizing cooperative game theory to provide insights into model predictions [26]. The SHAP value quantifies the contribution of each feature in an individual sample to the target outcome. These contributions can vary across different samples for the same feature, with the final prediction of the model for a sample being the sum of the SHAP values for all its features. For detailed calculations involving SHAP, please refer to Text S3.

### Dust composite imagery

Monitoring large-scale dust storms is highly challenging, and geostationary satellite imagery serves as an effective tool for comprehensively tracking the genesis, development and dissipation of these events. To verify the reliability of the gapless PM_10_ product retrieved by the RT-SPMR model, we utilized hourly composite dust imagery from the Himawari-8 satellite. This imagery is generated primarily from brightness temperatures at wavelengths of 12.4, 10.4 and 8.6 µm, which are rendered into RGB channels through difference calculations. The RGB composite dust imagery provides a more effective method for dust storm monitoring compared with various dust-related parameters by visually depicting the spatial distribution and dynamic evolution of dust storms, and offering real-time monitoring capabilities. To enhance the visibility of dust phenomena, gamma value correction is applied to intensify the high/low brightness pixel values. For detailed descriptions of the RGB composite dust scheme and image enhancement calculations, refer to Gui *et al.* [1].

## Supplementary Material

nwae446_Supplemental_File
