# Supplementary material for: Real-time mapping of gapless 24-hour surface PM10 in China
Source: Natl Sci Rev. 2024 Dec 9;12(2):nwae446. doi: 10.1093/nsr/nwae446 (PMC11925011; doi:10.1093/nsr/nwae446)
Supplement: nwae446_Supplemental_File [file nwae446_supplemental_file.docx]

***Supplementary Information for***

**Real-time mapping of gapless 24-hour surface PM_10_ in China**

Xutao Zhang^1, #^, Ke Gui^1, #,*^, Hengheng Zhao^1^, Nanxuan Shang^1^, Zhaoliang Zeng^2^, Wenrui Yao^1^, Lei Li^1^, Yu Zheng^1^, Hujia Zhao^3^, Yurun Liu^1,4^, Yucong Miao^1^, Yue Peng^1^, Ye Fei^5^, Fugang Li^6,7^, Baoxin Li^6,7^, Hong Wang^1^, Zhili Wang^1^, Yaqiang Wang^2^, Huizheng Che^1, *^, Xiaoye Zhang^1^

^1^State Key Laboratory of Severe Weather & Key Laboratory of Atmospheric Chemistry of CMA, Chinese Academy of Meteorological Sciences, Beijing, 100081, China

^2^ Institute of Artificial Intelligence for Meteorological, Chinese Academy of Meteorological Sciences, Beijing 100081, China

^3^ Institute of Atmospheric Environment, China Meteorological Administration, Shenyang 110166, China

^4^ Plateau Atmospheric and Environment Key Laboratory of Sichuan Province, College of Atmosphere Sciences, Chengdu University of Information Technology, Chengdu 610225, China

^5^ National Meteorological Information Center, Beijing 100081, China

^6^ China Global Atmosphere Watch Baseline Observatory, Xining 810001, China

^7^ Greenhouse Gas and Carbon Neutral Key Laboratory of Qinghai Province, Xining 810001, China

# These authors contributed equally

* Corresponding authors.

E-mail addresses: guik@cma.gov.cn (K. Gui) and chehz@cma.gov.cn (H. Che)

**This document includes Supplementary Text S1–S3, Supplementary Algorithm S1, Supplementary Tables 1–3, and Supplementary Figures 1–12.**

**Supplementary Text S1: Description, processing, and integration of multisource data**

Hourly surface PM_10_ observations from approximately 1800 stations were collected from the China National Environmental Monitoring Center network. To improve data quality, we performed quality control on the PM_10_ observation time series from each station. Specifically, we excluded data points that were identical for three consecutive hours and removed values that exceeded three standard deviations from the 24-hour moving average [1,2]. Unlike previous studies that widely used AOD, this study employed gridded SV data as the key intermediate variable for retrieving PM_10_. SV, which represents near-surface horizontal atmospheric transparency, has demonstrated notable potential in estimating aerosol-related parameters such as AOD and PM_2.5_ on both global and regional scales [2,3]. The gridded SV data originate from our newly developed SV retrieval framework [4], which operates in real-time and employs a two-layer stacked structure integrating various ML algorithms and deep learning modules [4]. By fusing multiple data sources, this framework can produce seamless hourly SV data with spatial resolution of 6.25 km for China. Overall, CV with observed data indicates that the SV data have high accuracy, with a correlation coefficient (*R*) of 0.95 and an RMSE of 3.17 km [4].

The surface meteorological fields were obtained from the CLDAS V2.0 at 0.0625° cell resolution [5]. CLDAS-V2.0 products are created by integrating surface meteorological observations, satellite products, and numerical model analysis and forecast data, utilizing multiple grid variational data assimilation techniques [6,7]. Specifically, six surface meteorological parameters were used in this study: 2-m temperature, surface pressure, 2-m RH, 10-m U- and V-wind components, and downward surface shortwave radiation.

To incorporate the influence of anthropogenic activities in our model, we used the 2020 population dataset from the Gridded Population of the World (GPW) version 4 (GPW-V4) [8], recalibrated to align with the total population reported in China City Yearbooks. We also included a monthly anthropogenic PM_10_ emission inventory for 2020 from the Multi-resolution Emission Inventory for China (MEIC) [9,10]. GPW-V4 has spatial resolution of 30 arcseconds, while the PM_10_ emission data have resolution of 0.25° × 0.25°. Additionally, we integrated sand fraction data from the Harmonized World Soil Database (HWSD) version 1.2 (with spatial resolution of 0.05° × 0.05°) [11], and monthly normalized difference vegetation index (NDVI) data for 2020 retrieved by the Moderate Resolution Imaging Spectroradiometer (spatial resolution: 250 m). These datasets help account for the effects of monthly subsurface changes and identify the primary dust sources in China. To capture the inherent spatiotemporal patterns of PM_10_ evolution on different time scales, we also incorporated several spatiotemporal features into the model, including elevation data at 300-m spatial resolution from 2-min Gridded Global Relief Data (ETOPO2), in addition to temporal features (month, day, and hour) and spatial features (longitude and latitude).

**Supplementary Text S2: Development, validation, and application of the RT-SPMR Framework**

In the data processing phase (step 1 in Fig. 1), we first apply the inverse distance weighting method to uniformly interpolate all predictors (except for population, which is summed within a 0.0625° × 0.0625° grid) to spatial resolution of 0.0625° × 0.0625°, consistent with SV and meteorological fields, thereby forming a multivariate feature matrix dataset. For details on the method for retrieval and processing of the gridded SV data, refer to Zhang et al. [4]. Subsequently, based on geographic location (latitude and longitude) and observation time, we use the nearest neighbor interpolation method to perform spatiotemporal sampling of the multivariate feature matrix at each PM_10_ observation site, creating a comprehensive multisite, multivariate time series from 2020 to 2022.

In the model construction phase (step 2 in Fig. 1), we introduced the AutoML technique to determine the optimal PM_10_ retrieval model. This study evaluated six ML models: CatBoost (categorical boosting), XGBoost (eXtreme Gradient Boosting), LightGBM (Light Gradient Boosting Machine), RandomForest, ExtraTrees, and MLP (Multilayer Perceptron). These models are well established in the field of quantitative retrieval of atmospheric pollutants and are known for their high accuracy [12–14]. However, given the requirements for real-time operations, we did not select a model arbitrarily; instead, we designed a comprehensive AutoML testing process (see Algorithm S1) to balance accuracy and computational efficiency. Specifically, we used a training set from 2020–2021 to explore different ML models and their parameter pools, thereby obtaining various model states. We then calculated the RMSE for these states using the 2022 validation set to evaluate performance. Both the RMSE results and the computational time costs for each model state were recorded. Table S1 provides a detailed record of our process for finding the optimal model by tuning the core parameters of each model. Upon completing the training of all parameters and models, we selected the model with the shortest training time and the lowest RMSE as the benchmark for the RT-SPMR model.

In the model application phase (step 3 in Fig. 1), we employed a rolling training method to continuously optimize the model determined in step 2. Specifically, we used a sliding window of one–two years of data to train the model, which was then applied for the 24-hour PM_10_ retrieval of the subsequent day. This iterative process allows the model to continuously adapt to the latest data, enhancing its accuracy and stability in reflecting recent environmental changes and pollution trends. To achieve this, we deployed data reception and processing modules on a cloud platform for automated operation. Once all real-time updated data are received, the cloud platform automatically processes these data, together with inherent auxiliary information, ensuring that all data reach a uniform grid resolution of 6.25 km, thereby forming the input prediction matrix. Through this process, the cloud platform can autonomously complete the iterative updating of the RT-SPMR model and perform daily PM_10_ retrieval tasks. Currently, all input features are updated in real-time with a delay of approximately 40 min, allowing us to produce hourly gapless PM_10_ products.

**Supplementary Text S3:** **Calculating feature contributions using the SHAP approach**

For an individual sample in the dataset, the target value (PM_10_ in this case) can be decomposed as follows:

$$\begin{aligned} f\left( x \right)=\phi_{0}+\sum_{i=1}^{M} \phi_{i}z_{i}^{'} \end{aligned}\left( 1 \right)$$

where $f\left( x \right)$ is the target value, $M$ is the total number of features, $\phi_{0}$ and $\phi_{i}$ represent the baseline SHAP value and the SHAP value of each feature, respectively, and $z_{i}^{'}\in{\{0, 1\}}^{M}$ represents the number of features in the decision path of the model. For a given sample, the SHAP value of feature $i (i\in\left[ 1,M \right])$ is computed using the following formula:

$$\begin{aligned} \phi_{i}=\sum_{S\subseteq G\backslash\{i\}} \frac{\left| S \right|!\left( M-\left\{ S \right\}-1 \right)!}{M!}\left[ f_{\chi}\left( S\cup\left\{ i \right\} \right)-f_{\chi}\left( S \right) \right] \end{aligned}\left( 2 \right)$$

where $G$ is the set of all input features, $S$ is the set of non-zero indices in $z_{i}^{'}$, and $f_{\chi}(S)$ is the expected value of the function conditioned on a subset $S$ of the input features. Thus, utilizing the SHAP values for each feature in each sample, we can determine the overall feature contribution for a region:

$$\begin{aligned} \varphi_{i}=\frac{\underset{k=1}{\overset{N}{\sum}}|\phi_{ik}|}{N} \end{aligned}\left( 3 \right)$$

where $\varphi_{i}(i\in\left[ 1,M \right])$represents the single-feature importance of the selected dataset and $N$ represents the total number of samples used.

**Algorithm S1.** Modelling process for automated machine learning.


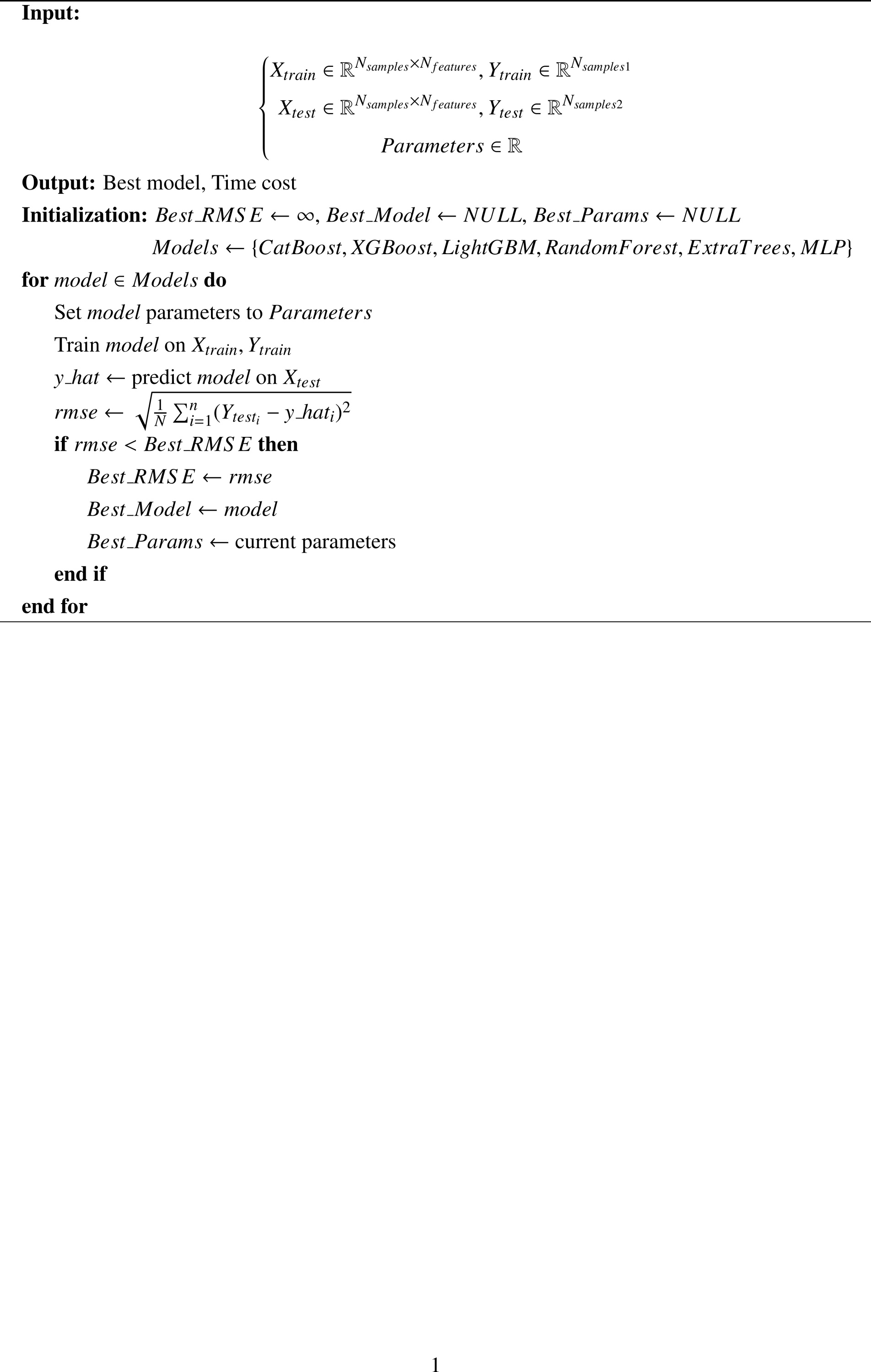


**Table S1.** Predictive performance of different ML models under different parameter settings in the hindcast validation experiment.

| Model | Parameters | | | | Results | | |
| --- | --- | --- | --- | --- | --- | --- | --- |
| CatBoost | Learning rate | Max depth | | Growing policy | *R*^2^ | RMSE (μg·m^−3^) | Time cost^a^ (s) |
|  | 0.01 | 8 | | Symmetric Tree | 0.65 | 36.93 | 307.78 |
|  | 0.01 | 8 | | Depth Wise | 0.66 | 36.33 | 111.61 |
|  | 0.01 | 12 | | Symmetric Tree | 0.68 | 35.66 | 447.52 |
|  | 0.01 | 12 | | Depth Wise | 0.66 | 36.59 | 102.42 |
|  | 0.01 | 16 | | Symmetric Tree | **0.69**^b^ | **35.10** | **490.38** |
|  | 0.01 | 16 | | Depth Wise | 0.64 | 37.54 | 330.85 |
|  | 0.02 | 8 | | Symmetric Tree | 0.65 | 36.85 | 162.78 |
|  | 0.02 | 8 | | Depth Wise | 0.65 | 37.30 | 25.66 |
|  | 0.02 | 12 | | Symmetric Tree | 0.67 | 36.05 | 155.65 |
|  | 0.02 | 12 | | Depth Wise | 0.66 | 36.40 | 51.26 |
|  | 0.02 | 16 | | Symmetric Tree | 0.68 | 35.29 | 271.64 |
|  | 0.02 | 16 | | Depth Wise | 0.64 | 37.33 | 181.71 |
|  | 0.05 | 8 | | Symmetric Tree | 0.65 | 36.95 | 66.24 |
|  | 0.05 | 8 | | Depth Wise | 0.66 | 36.41 | 26.89 |
|  | 0.05 | 12 | | Symmetric Tree | 0.67 | 35.96 | 79.98 |
|  | 0.05 | 12 | | Depth Wise | 0.66 | 36.45 | 54.78 |
|  | 0.05 | 16 | | Symmetric Tree | 0.68 | 35.33 | 129.39 |
|  | 0.05 | 16 | | Depth Wise | 0.66 | 36.77 | 150.17 |
| XGBoost | Learning rate | | Max depth | | *R*^2^ | RMSE (μg·m^−3^) | Time cost (s) |
|  | 0.01 | | 16 | | 0.63 | 38.80 | 334.26 |
|  | 0.01 | | 18 | | 0.63 | 39.19 | 901.74 |
|  | 0.01 | | 20 | | 0.62 | 39.81 | 1636.59 |
|  | 0.01 | | 22 | | 0.60 | 40.40 | 4554.16 |
|  | 0.02 | | 16 | | 0.63 | 38.97 | 196.30 |
|  | 0.02 | | 18 | | 0.63 | 38.96 | 477.17 |
|  | 0.02 | | 20 | | 0.62 | 39.63 | 949.99 |
|  | 0.02 | | 22 | | **0.63** | **38.65** | **2866.59** |
|  | 0.05 | | 16 | | 0.64 | 39.68 | 92.14 |
|  | 0.05 | | 18 | | 0.63 | 39.23 | 198.28 |
|  | 0.05 | | 20 | | 0.61 | 40.37 | 422.31 |
|  | 0.05 | | 22 | | 0.61 | 40.27 | 1034.25 |
| LightGBM | Learning rate | | Max depth | | *R*^2^ | RMSE (μg·m^−3^) | Time cost (s) |
|  | 0.01 | | 19 | | 0.68 | 35.67 | 1191.06 |
|  | 0.01 | | 23 | | 0.67 | 35.73 | 1001.93 |
|  | 0.01 | | 25 | | 0.67 | 35.73 | 1081.27 |
|  | 0.02 | | 19 | | **0.68** | **35.69** | **723.44** |
|  | 0.02 | | 23 | | 0.68 | 35.70 | 627.72 |
|  | 0.02 | | 25 | | 0.67 | 35.73 | 494.13 |
|  | 0.05 | | 19 | | 0.67 | 35.73 | 266.38 |
|  | 0.05 | | 23 | | 0.67 | 35.87 | 212.63 |
|  | 0.05 | | 25 | | 0.67 | 35.78 | 229.10 |
| RandomForest | Estimators | | Max depth | | *R*^2^ | RMSE (μg·m^−3^) | Time cost (s) |
|  | 80 | | 17 | | 0.59 | 39.92 | 5482.86 |
|  | 80 | | 19 | | 0.60 | 39.46 | 5970.85 |
|  | 80 | | 21 | | 0.58 | 40.79 | 6581.10 |
|  | 110 | | 17 | | 0.61 | 39.18 | 7116.27 |
|  | 110 | | 19 | | 0.62 | 38.55 | 7774.68 |
|  | 110 | | 21 | | 0.60 | 39.60 | 6131.81 |
|  | 130 | | 17 | | **0.63** | **38.84** | **4108.44** |
|  | 130 | | 19 | | 0.62 | 38.58 | 9598.45 |
|  | 130 | | 21 | | 0.61 | 39.15 | 7770.09 |
| ExtraTrees | Estimators | | Max depth | | *R*^2^ | RMSE (μg·m^−3^) | Time cost (s) |
|  | 80 | | 17 | | 0.65 | 37.15 | 1174.96 |
|  | 80 | | 19 | | 0.65 | 36.85 | 1191.04 |
|  | 80 | | 21 | | 0.66 | 36.58 | 1054.51 |
|  | 110 | | 17 | | 0.65 | 37.08 | 1257.91 |
|  | 110 | | 19 | | 0.66 | 36.76 | 1240.68 |
|  | 110 | | 21 | | 0.66 | 36.53 | 1319.21 |
|  | 130 | | 17 | | 0.65 | 37.02 | 1388.87 |
|  | 130 | | 19 | | **0.67** | **36.49** | **1357.78** |
|  | 130 | | 21 | | 0.66 | 36.53 | 1623.43 |
| MLP | Hidden layers^b^ | | | | *R*^2^ | RMSE (μg·m^−3^) | Time cost (s) |
|  | (64, 128, 256, 128, 64) | | | | 0.62 | 38.50 | 3214 |
|  | (64, 128, 256, 256, 128, 64) | | | | 0.64 | 37.90 | 4211 |
|  | (64, 128, 256, 512, 256, 128, 64) | | | | **0.65** | **37.48** | **5173** |
|  | (64, 128, 256, 512, 512, 256, 128, 64) | | | | 0.63 | 38.20 | 6722 |

^a^All experiments were conducted on a single GeForce RTX 3090 GPU and 24-threaded Intel i9-12900K CPUs.

^b^In table S1, bold text indicates the optimal parameters for each model.

^c^The number in parentheses represents the number of hidden layers, while the number itself represents the number of neurons. All MLP models use the Rectified Linear Unit (ReLu) activation function.

**Table S2.** Performance of the RT-SPMR model in terms of spatial/temporal resolution, key predictor, cross-validation (CV) results, spatial coverage, and updating capability, compared with other national-scale PM_10_ retrieval studies in China.

| Study | Spatial  resolution | Key  predictor | Study  period | Temporal  resolution | *R*^2^ | | RMSE  (μg·m^−3^) | | Real-time update capability | Seamless |
| --- | --- | --- | --- | --- | --- | --- | --- | --- | --- | --- |
|  |  |  |  |  | Sample-based  CV | Site-based  CV | Sample-based  CV | Site-based  CV |  |  |
| Chen et al. [15] | 0.1° × 0.1° | AOD | 2005–2016 | Daily | 0.78  (10-fold) | — | 31.5  (10-fold) | — | No | No |
| Zhang et al. [16] | 0.1° × 0.1° | AOD | 2014–2016 | Monthly | 0.64  (10-fold) | — | — | — | No | No |
| Wei et al. [17] | 0.01° × 0.01° | AOD | 2015–2019 | Daily | 0.86  (10-fold) | 0.82  (10-fold) | 24.3  (10-fold) | 27.1  (10-fold) | No | Yes |
| Chen et al.[18] | 0.04° × 0.04° | TOA^a^ | 2018–2019 | Hourly  (daytime) | — | 0.66  (10-fold) | — | 31.84  (10-fold) | No | No |
|  |  |  |  | Daily | 0.82  (10-fold) | — | 24.16  (10-fold) | — |  |  |
| Chen et al.[19] | 0.05° × 0.05° | AOD | 2018–2019 | Hourly  (daytime) | 0.82–0.88  (10-fold) | — | 18.6–23.1  (10-fold) | — | No | No |
|  |  |  |  | Daily | 0.87  (10-fold) | — | 19.4  (10-fold) | — |  |  |
| This study | 0.0625° × 0.0625° | SV | 2020–2022 | Hourly  (full 24-h) | 0.79  (5-fold) | 0.72  (5-fold) | 37.3  (5-fold) | 43.6  (5-fold) | Yes | Yes |
|  |  |  |  | Daily | 0.96  (5-fold) | 0.86  (5-fold) | 14.4  (5-fold) | 23.7  (5-fold) |  |  |

^a^Top of Atmosphere

**Table S3.** Summary of the data used in this study from multiple sources.

| Data  category | Data  name | Spatial  resolution | Temporal frequency | Time  coverage | Data  source/reference | Updated  lag |
| --- | --- | --- | --- | --- | --- | --- |
| Ground observations | PM_10_ measurements | Point | Hourly | 2020 to Date | https://air.cnemc.cn:18007/ | ~30 min |
| Gridded SV  fields | SV | 0.0625° × 0.0625° | Hourly | 2020 to Date | https://doi.org/10.1038/s41612-024-00617-1 | ~35 min |
| Meteorological fields | CLDAS-V2.0 | 0.0625° × 0.0625° | Hourly | 2020 to Date | http://data.cma.cn/data/cdcdetail/dataCode/NAFP_CLDAS2.0_RT.html | ~40 min |
| Population | GPW-V4 | 30 arcseconds | Yearly | 2020 | https://beta.sedac.ciesin.columbia.edu | - |
| Elevation | ETOPO2 | 300 m | - | - | https://www.ncei.noaa.gov/products/etopo-global-relief-model | - |
| PM_10_ emission | MEIC | 0.25° × 0.25° | Monthly | 2020 | http://meicmodel.org | - |
| NDVI | MYD13Q1 | 250 m | Monthly | 2020 | https://lpdaac.usgs.gov/products/myd13q1v006/ | 1 month |
| Sand fraction | HWSD-V1.2 | 0.05° × 0.05° | - | - | https://daac.ornl.gov/cgi-bin/dsviewer.pl?ds_id=1247 | - |


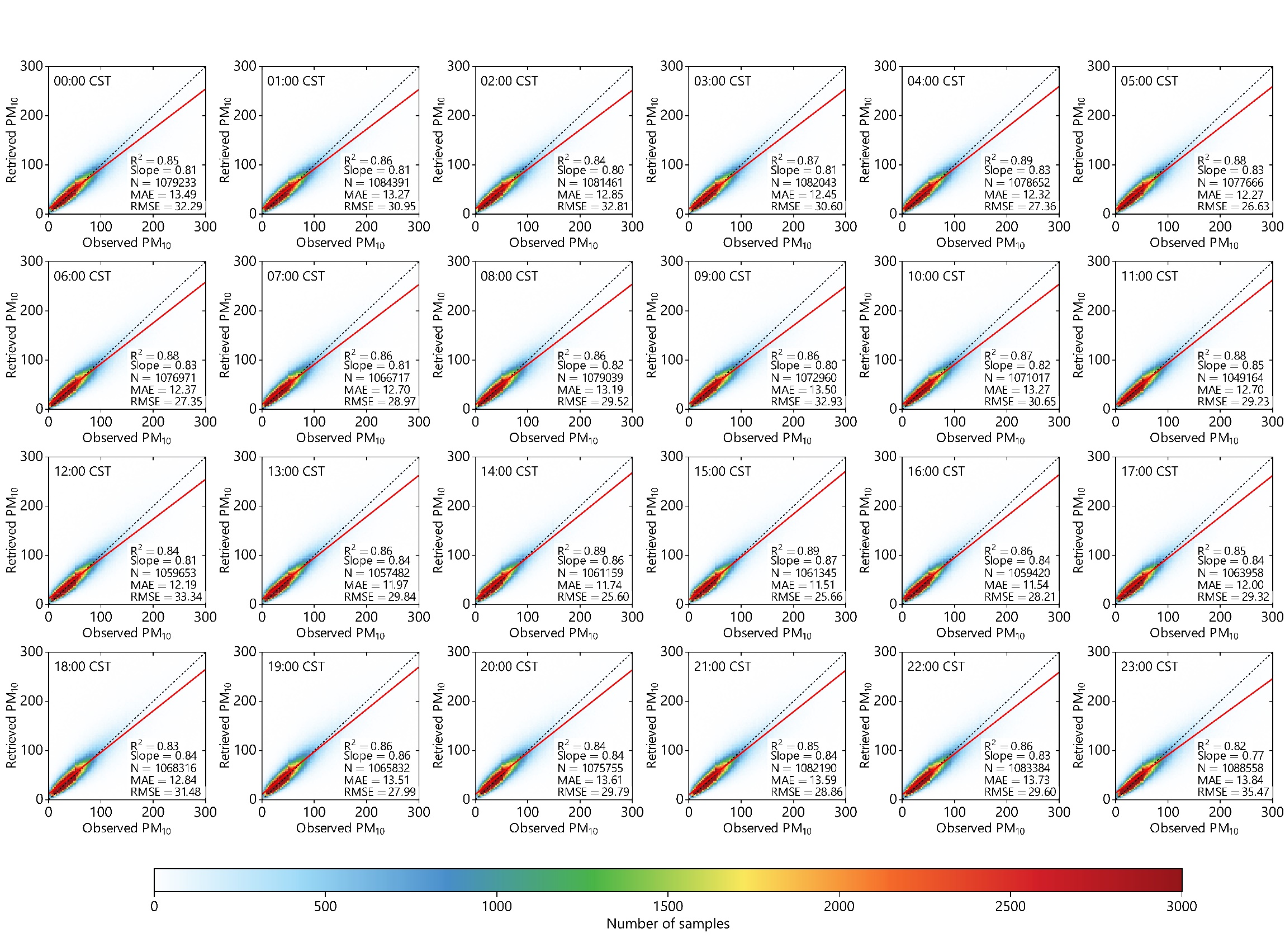


**Figure S1**. Density scatterplots between observed hourly PM_10_ and estimated hourly PM_10_ across China for the sample-based 5-fold CV during 2020–2021. Note that these CV results are calculated on an hourly basis and grouped by each specific hour.


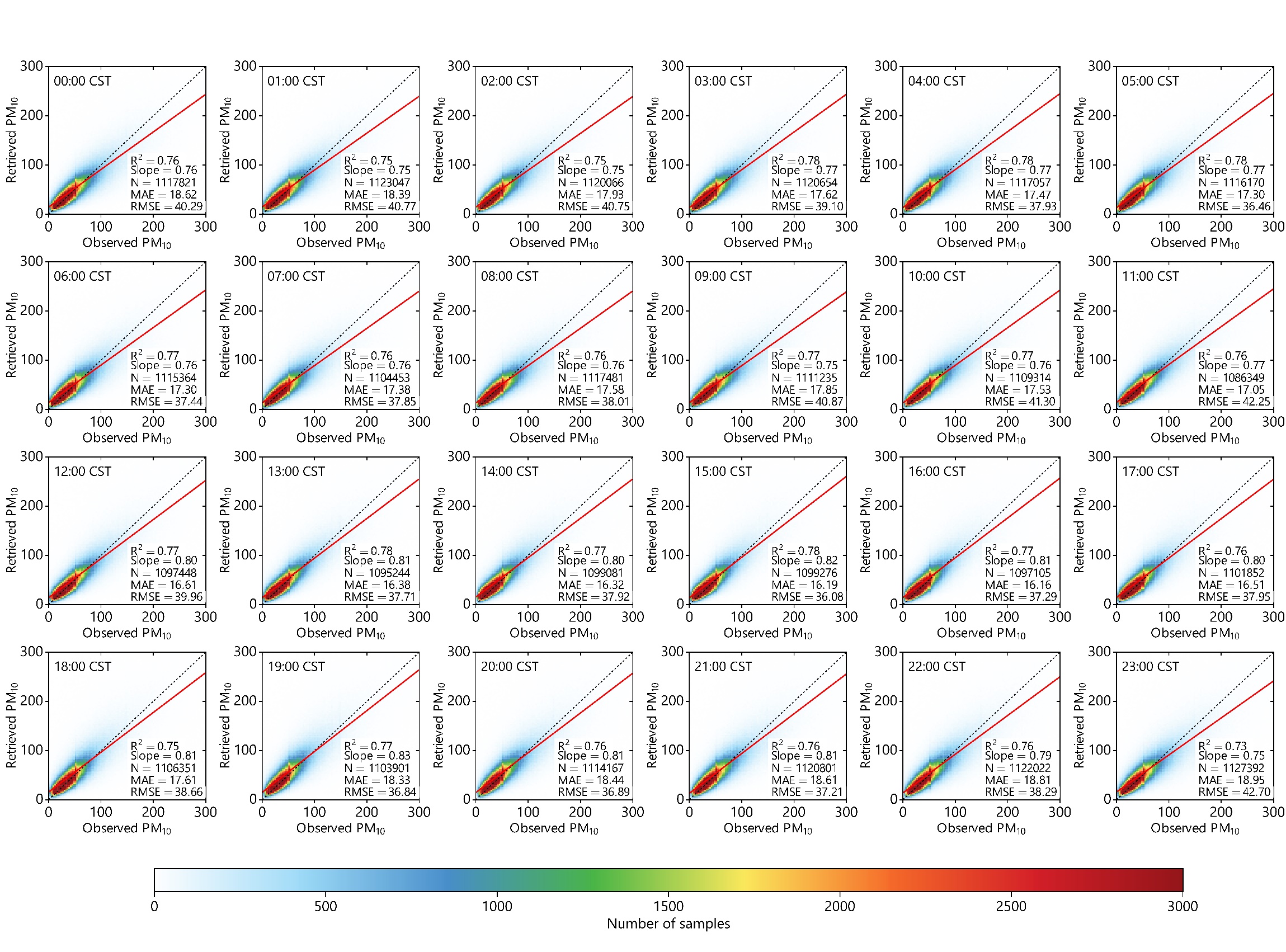


**Figure S2.** Same as Fig. S1 but for the statistical results of the site-based CV during 2020–2021. Note that these CV results are calculated on an hourly basis and grouped by each specific hour.


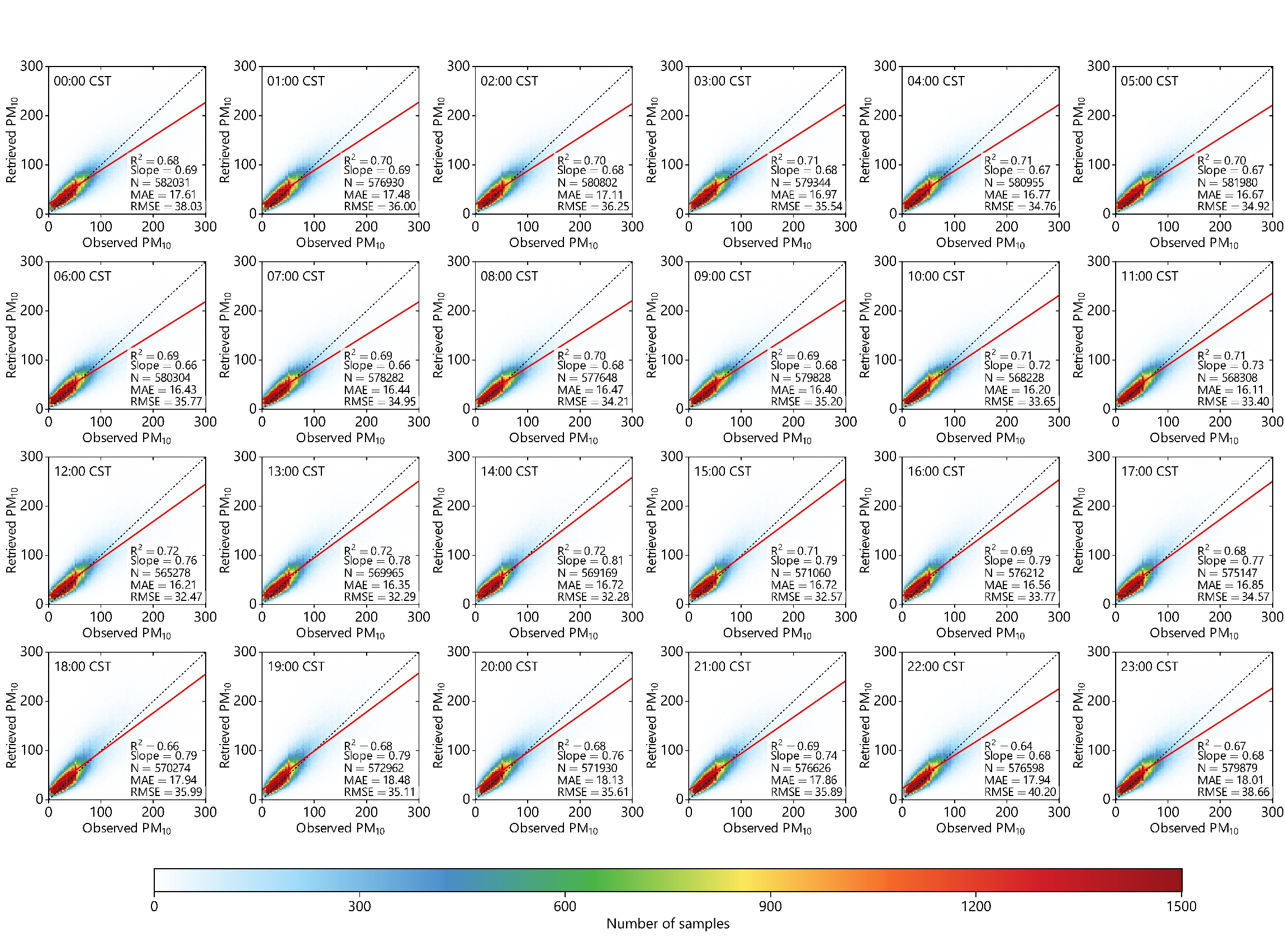


**Figure S3.** Same as Fig. S1 but for the statistical results of the HV experiment in 2022. Note that these HV results are calculated on an hourly basis and grouped by each specific hour.


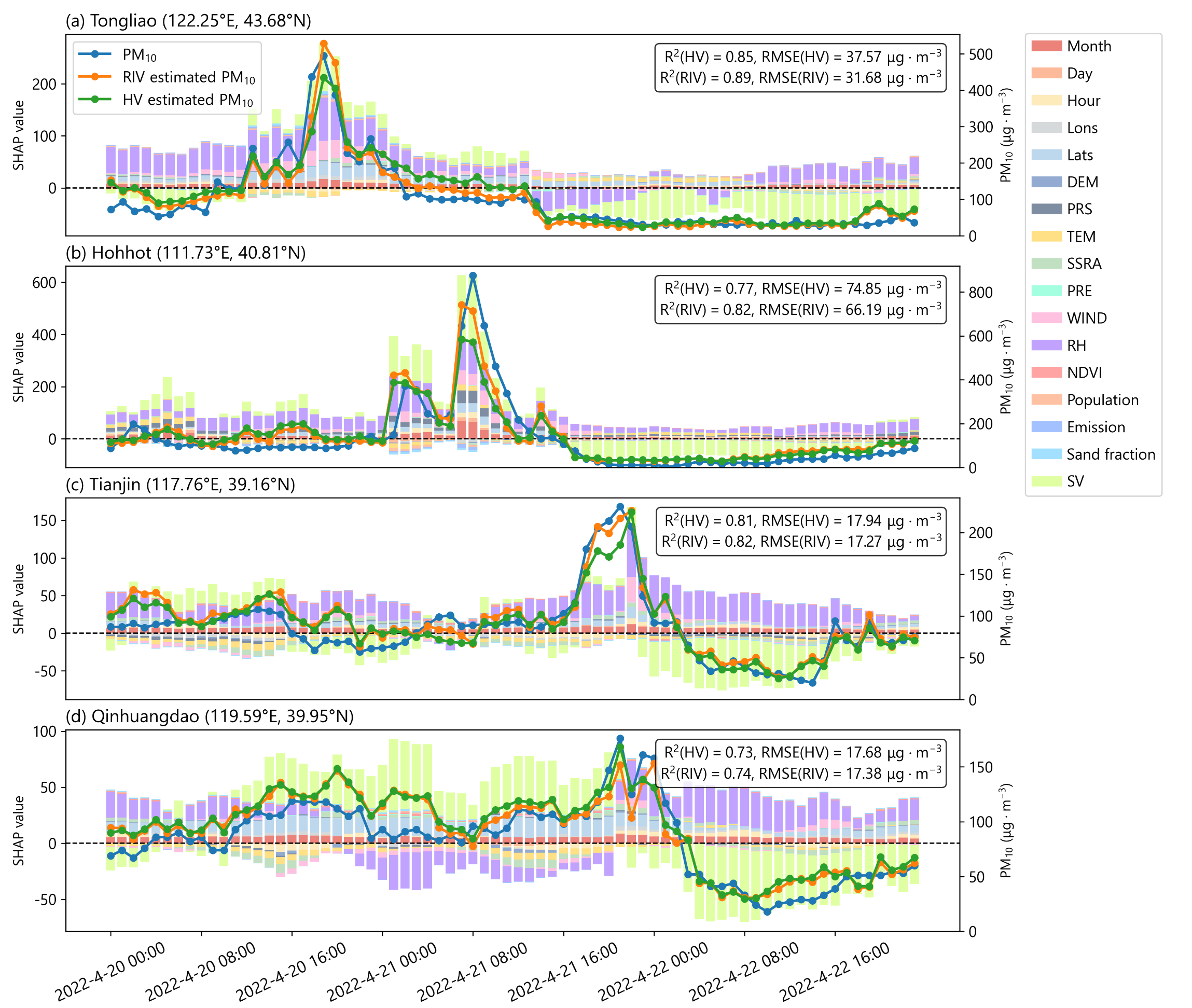


Figure S4. Hourly time series of observed PM_10_ concentrations, HV-estimated PM_10_, RIV-estimated PM_10_ concentrations, and SHAP values for each predictor at four typical sites, including (a) Tongliao, (b) Hohhot, (c) Tianjin, and (d) Qinhuangdao, during a typical dust event in northern China from April 20 to April 22, 2022.


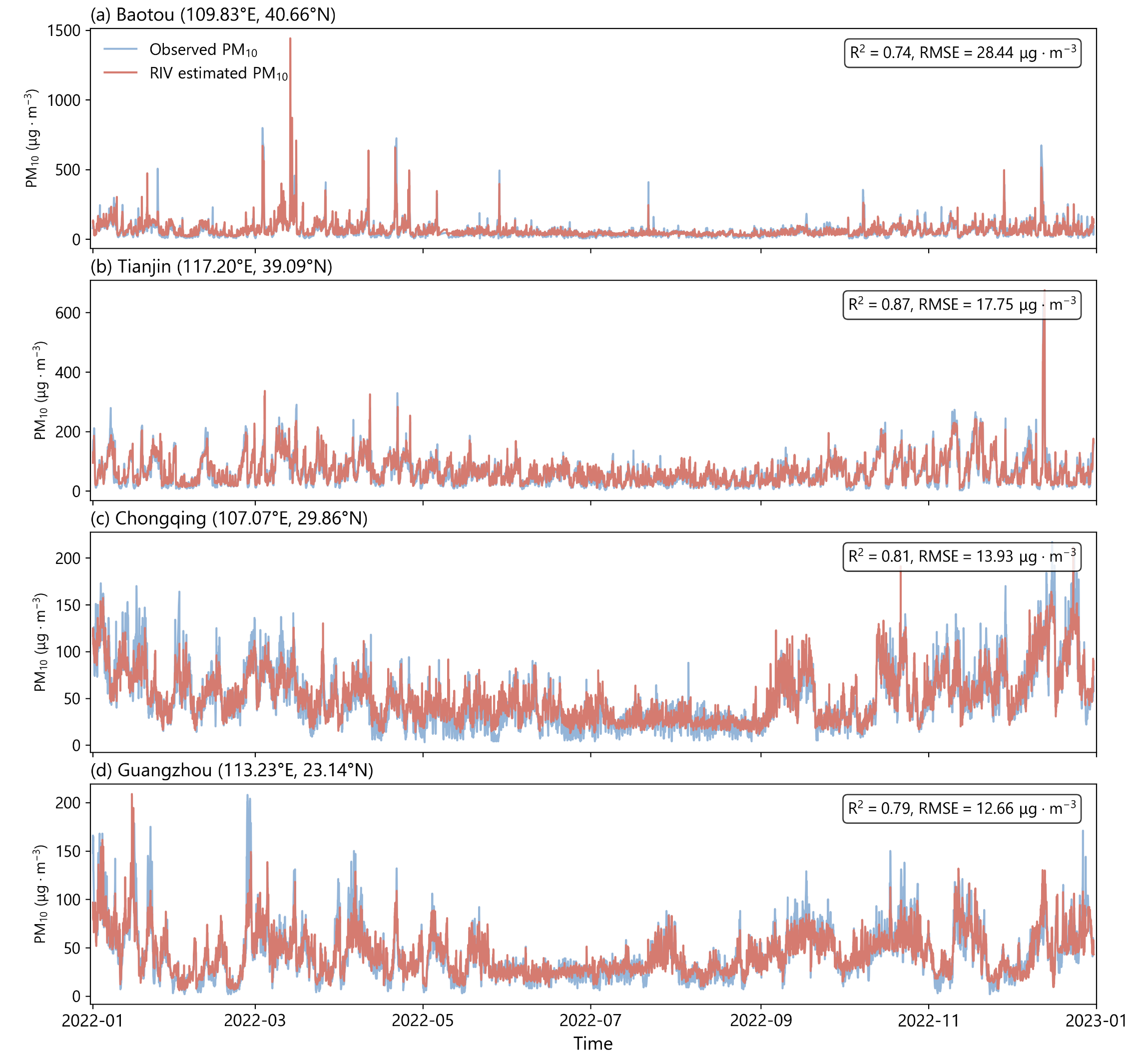


Figure S5. Hourly time series of observed and RIV-estimated PM_10_ concentrations for 2022 at four representative sites: (a) Baotou, (b) Tianjin, (c) Chongqing, and (d) Guangzhou.


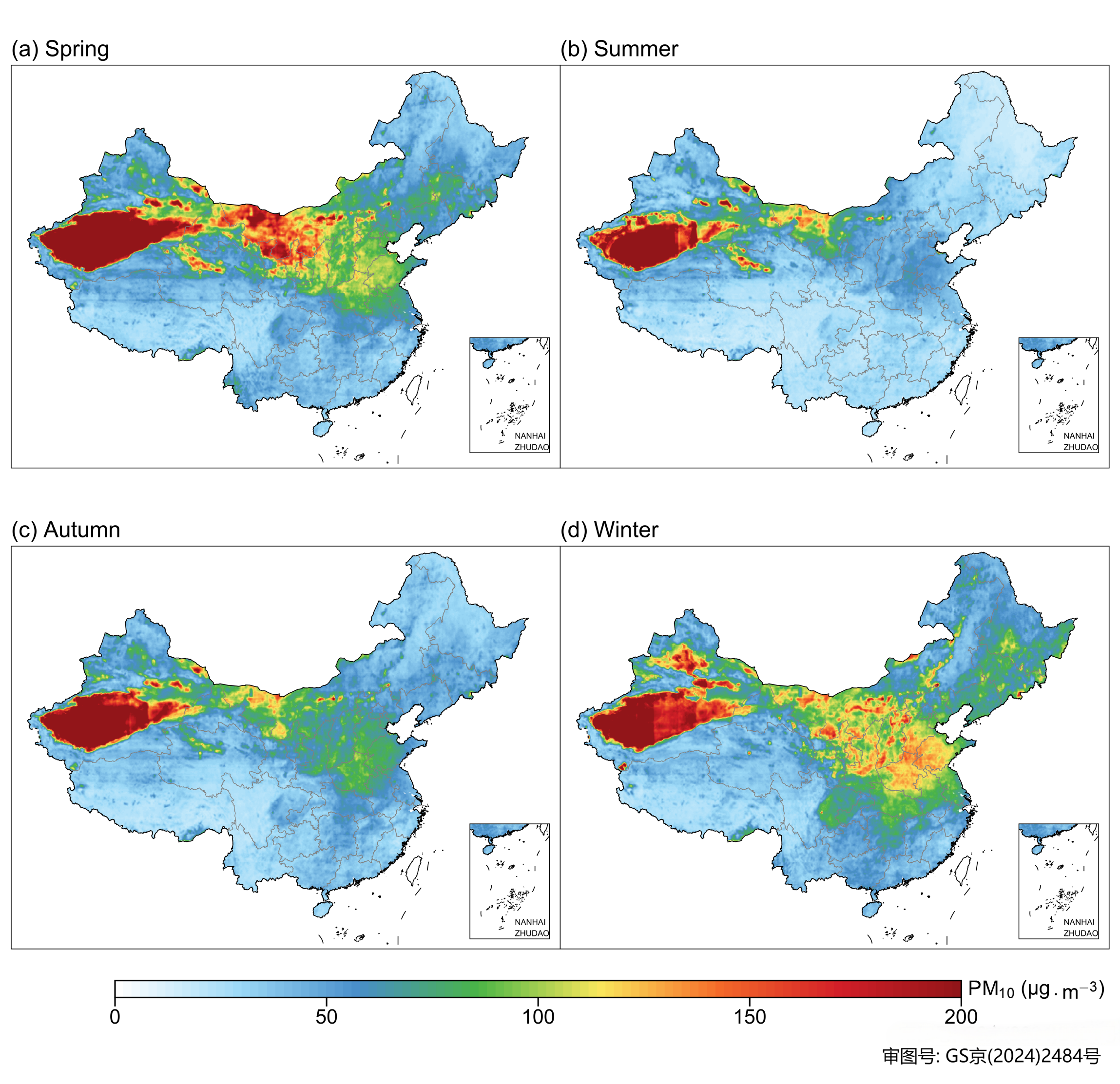


**Figure** **S6**. Multiyear averaged seasonal PM_10_ maps retrieved from the RT-SPMR model during 2020–2022.


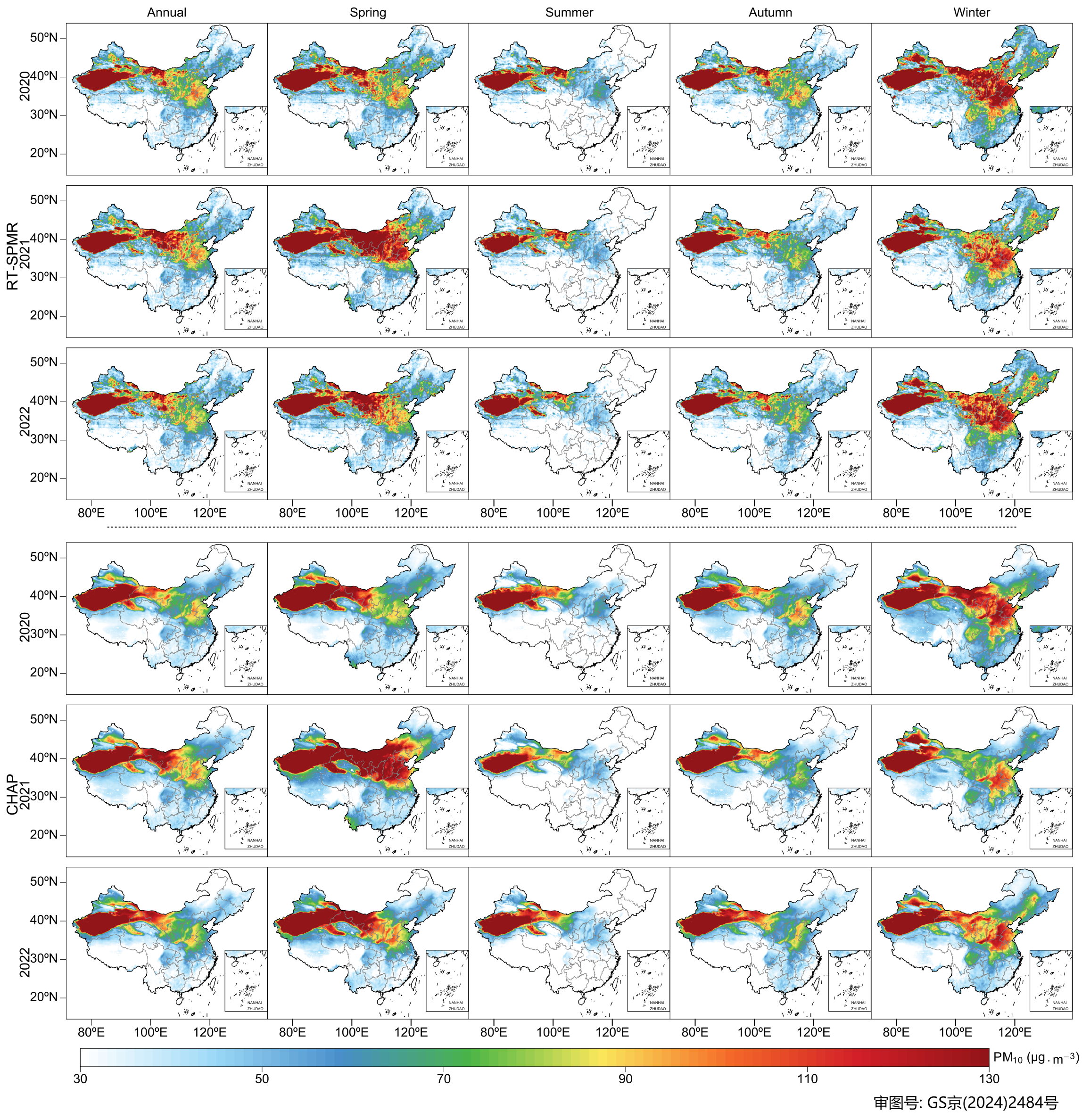


**Figure** **S7.** Comparison of annual and seasonal averages of PM_10_ retrievals between the RT-SPMR and ChinaHighAirPollutants (CHAP) datasets for 2020–2022. Note that for RT-SPMR and CHAP, the annual and seasonal averages are calculated on an hourly and daily basis, respectively.


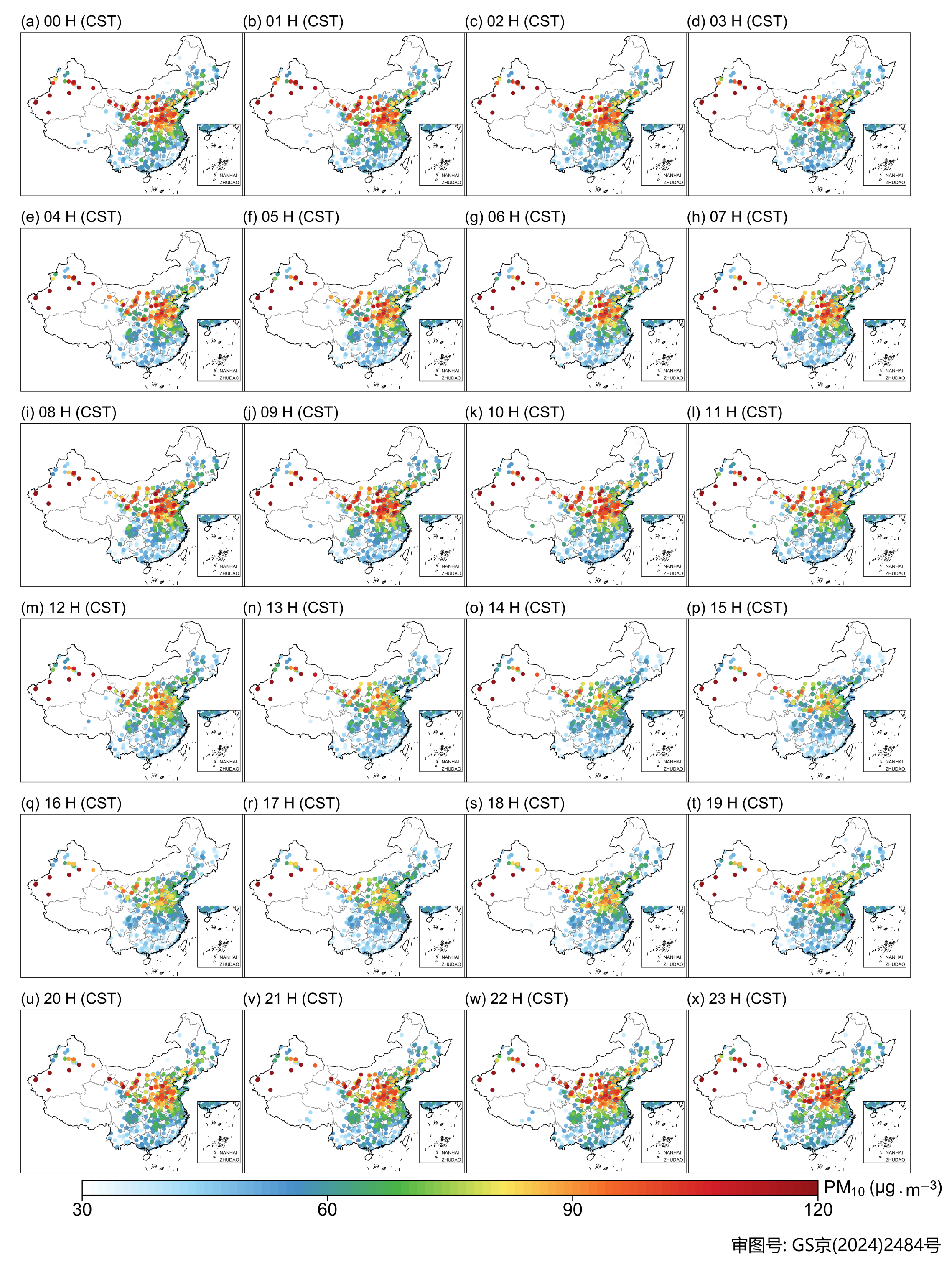


**Figure** **S8.** Diurnal cycle of observed PM_10_ concentrations for multiyear averages (2020–2022).


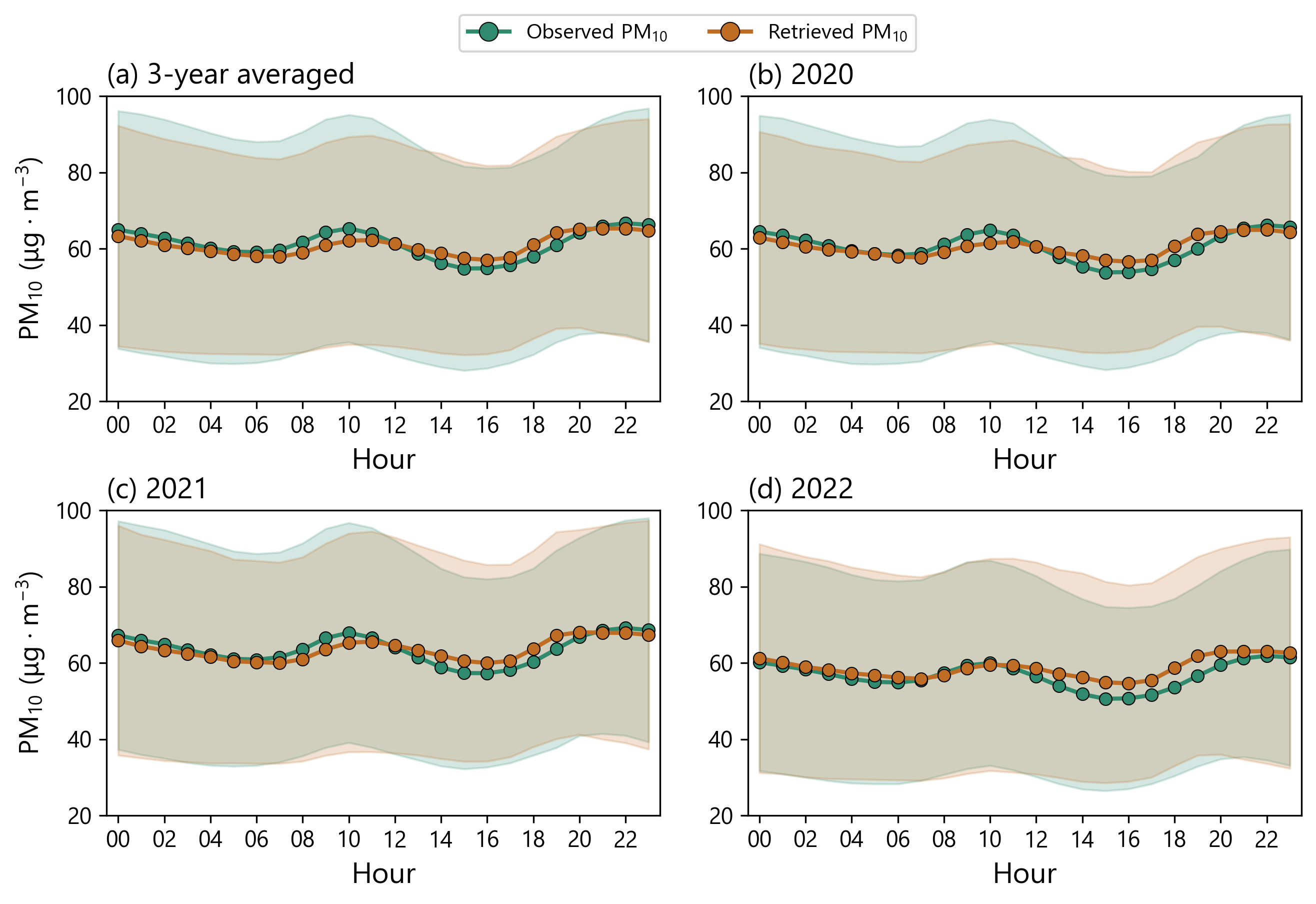


**Figure** **S9.** Comparison of diurnal patterns of regionally averaged hourly PM_10_ in China for (a) 2020–2022, (b) 2020, (c) 2021, and (d) 2022 between RT-SPMR retrievals and observations. Shaded areas indicate standard deviations.

**
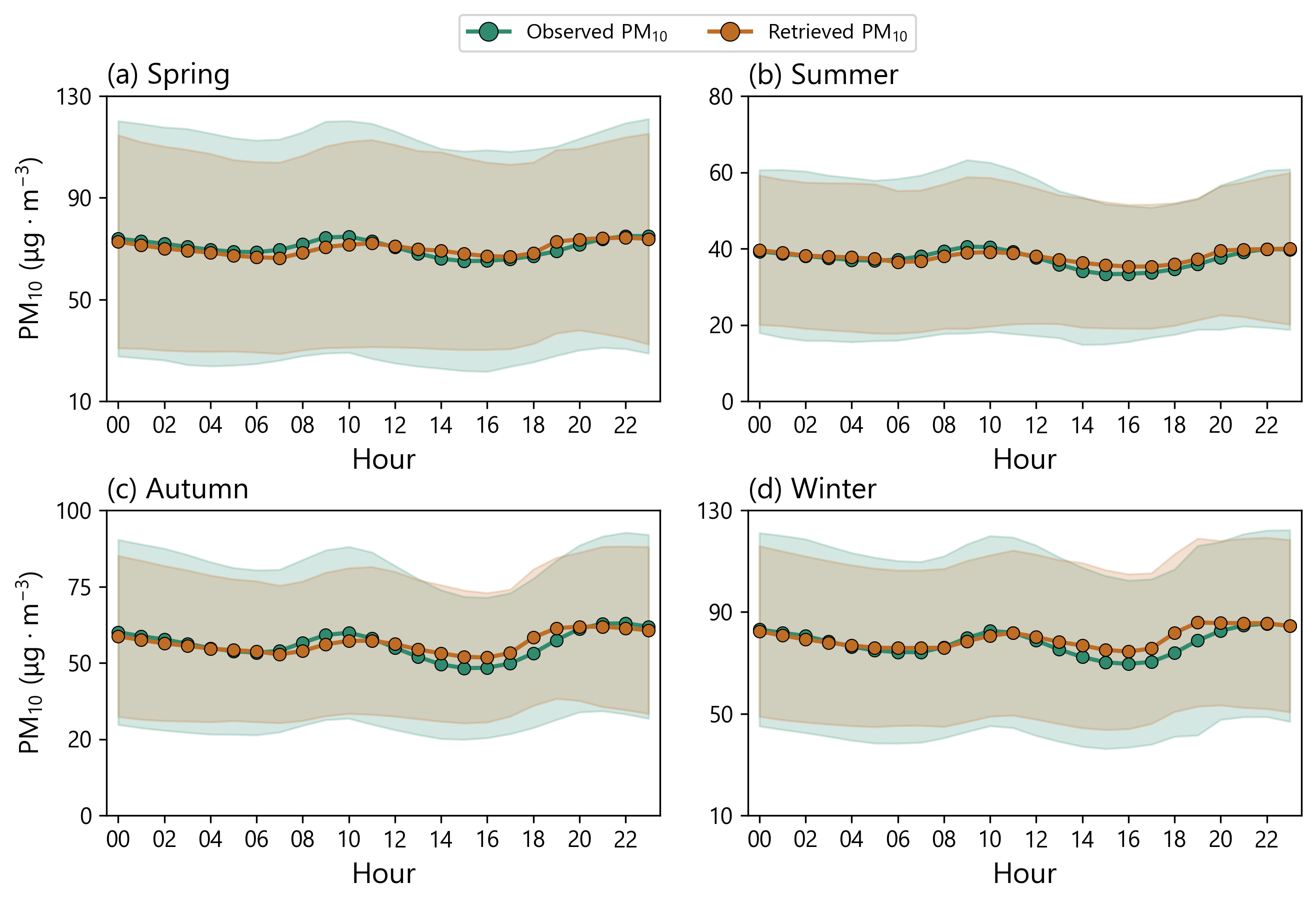
**

**Figure S10.** Comparison of diurnal patterns of regionally averaged hourly PM_10_ in China for 2020–2022 during (a) spring, (b) summer, (c) autumn, and (d) winter, between RT-SPMR retrievals and observations. Shaded areas indicate standard deviations. Note that the Y-axis scale range varies by season.


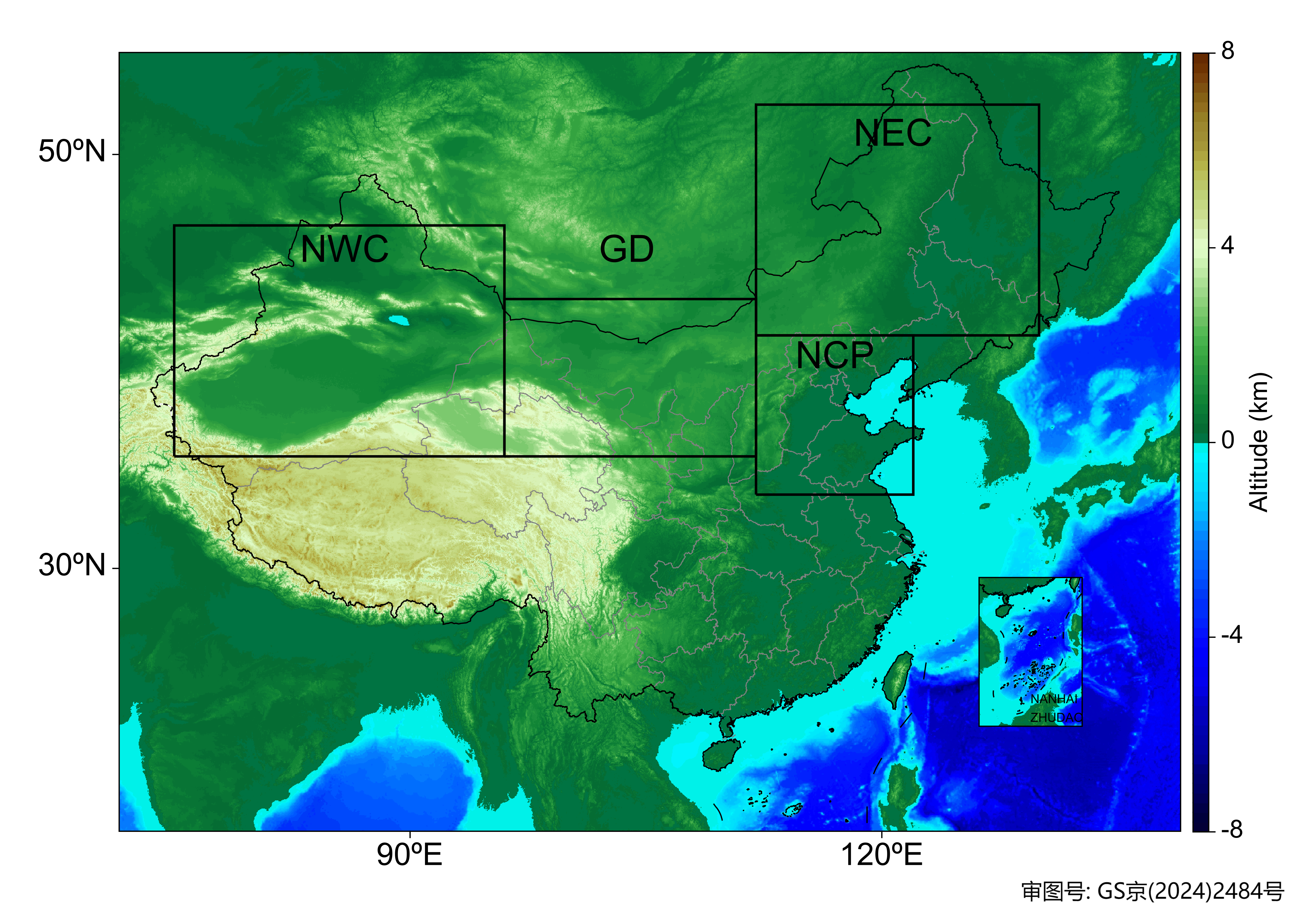


**Figure** **S11.** Geographic locations of the four subregions used in this study: Northwest China (NWC; 36–47°N, 75–96°W), Gobi Desert (GD; 36–47°N, 96–112°W), Northeast China (NEC; 42–52°N, 112–130°W), and the North China Plain (NCP; 34–42°N, 112–122°W).


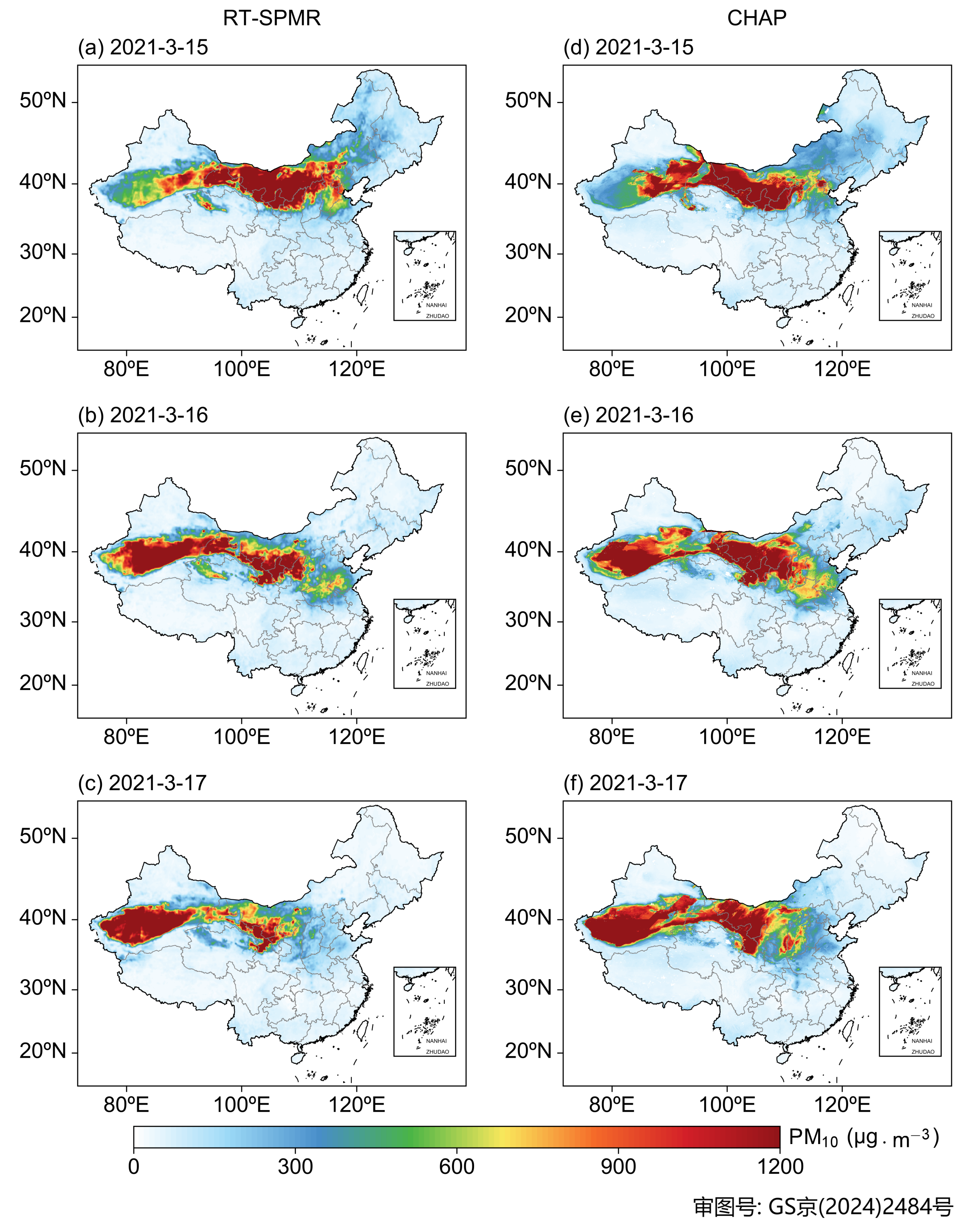


**Figure** **S12.** Distribution of daily PM_10_ concentrations retrieved from (left) the RT-SPMR model and (right) the CHAP dataset during 15–17 March 2021. Note that for RT-SPMR, the daily PM_10_ concentration is calculated on an hourly basis.

**References**

1. Yan X, Zuo C, Li Z *et al.* Cooperative simultaneous inversion of satellite-based real-time PM_2.5_ and ozone levels using an improved deep learning model with attention mechanism. *Environ Pollut* 2023;**327**:121509.

2. Zhong J, Zhang X, Gui K *et al.* Robust prediction of hourly PM_2.5_ from meteorological data using LightGBM. *Natl Sci Rev* 2021;**8**, DOI: 10.1093/nsr/nwaa307.

3. Gui K, Che H, Zeng Z *et al.* Construction of a virtual PM_2.5_ observation network in China based on high-density surface meteorological observations using the Extreme Gradient Boosting model. *Environ Int* 2020;**141**:105801.

4. Zhang X, Gui K, Zeng Z *et al.* Mapping the seamless hourly surface visibility in China : a real-time retrieval framework using a machine-learning- based stacked ensemble model. *npj Clim Atmos Sci* 2024, DOI: 10.1038/s41612-024-00617-1.

5. Shi C, Jiang L, Zhang T *et al.* Status and Plans of CMA Land Data Assimilation System (CLDAS) Project. *EGU Gen Assem Conf Abstr* 2014;**16**:5671.

6. Han S, Liu B, Shi C *et al.* Evaluation of CLDAS and GLDAS datasets for near-surface air temperature over major land areas of China. *Sustain* 2020;**12**, DOI: 10.3390/su12104311.

7. Shi C, Pan Y, Gu J *et al.* A review of multi-source meteorological data fusion products. *Acta Meteorol Sin* 2019;**77**:774–83.

8. Lloyd CT. High resolution global gridded data for use in population studies. *Int Arch Photogramm Remote Sens Spat Inf Sci - ISPRS Arch* 2017;**42**:117–20.

9. Li M, Liu H, Geng G *et al.* Anthropogenic emission inventories in China: A review. *Natl Sci Rev* 2017;**4**:834–66.

10. Zheng B, Tong D, Li M *et al.* Trends in China’s anthropogenic emissions since 2010 as the consequence of clean air actions. *Atmos Chem Phys* 2018;**18**:14095–111.

11. Saxton KE, Rawls WJ. Soil Water Characteristic Estimates by Texture and Organic Matter for Hydrologic Solutions. *Soil Sci Soc Am J* 2006;**70**:1569–78.

12. Wei J, Li Z, Lyapustin A *et al.* Reconstructing 1-km-resolution high-quality PM_2.5_ data records from 2000 to 2018 in China: spatiotemporal variations and policy implications. *Remote Sens Environ* 2021;**252**:112136.

13. Yang Q, Yuan Q, Li T *et al.* Mapping PM_2.5_ concentration at high resolution using a cascade random forest based downscaling model: Evaluation and application. *J Clean Prod* 2020;**277**:123887.

14. Geng G, Xiao Q, Liu S *et al.* Tracking Air Pollution in China: Near Real-Time PM_2.5_ Retrievals from Multisource Data Fusion. *Environ Sci Technol* 2021;**55**:12106–15.

15. Chen G, Wang Y, Li S *et al.* Spatiotemporal patterns of PM_10_ concentrations over China during 2005–2016: A satellite-based estimation using the random forests approach. *Environ Pollut* 2018;**242**:605–13.

16. Zhang Z, Wang J, Hart JE *et al.* National scale spatiotemporal land-use regression model for PM_2.5_, PM_10_ and NO_2_ concentration in China. *Atmos Environ* 2018;**192**:48–54.

17. Wei J, Li Z, Xue W *et al.* The ChinaHighPM_10_ dataset: generation, validation, and spatiotemporal variations from 2015 to 2019 across China. *Environ Int* 2021;**146**:106290.

18. Chen B, Song Z, Huang J *et al.* Estimation of Atmospheric PM_10_ Concentration in China Using an Interpretable Deep Learning Model and Top-of-the-Atmosphere Reflectance Data From China’s New Generation Geostationary Meteorological Satellite, FY-4A. *J Geophys Res Atmos* 2022;**127**:1–20.

19. Chen B, Song Z, Shi B *et al.* An interpretable deep forest model for estimating hourly PM_10_ concentration in China using Himawari-8 data. *Atmos Environ* 2022;**268**:118827.
